# A SpoIID Homolog Cleaves Glycan Strands at the Chlamydial Division Septum

**DOI:** 10.1128/mBio.01128-19

**Published:** 2019-07-16

**Authors:** Nicolas Jacquier, Akhilesh K. Yadav, Trestan Pillonel, Patrick H. Viollier, Felipe Cava, Gilbert Greub

**Affiliations:** a Institute of Microbiology, University Hospital Center and University of Lausanne, Lausanne, Switzerland; b Laboratory for Molecular Infection Medicine Sweden (MIMS), Department of Molecular Biology, Umeå University, Umeå, Sweden; c Department of Microbiology & Molecular Medicine, Institute of Genetics & Genomics in Geneva (iGE3), Faculty of Medicine/CMU, University of Geneva, Geneva, Switzerland; Duke University; University of California, Berkeley

**Keywords:** *Chlamydiales*, *Waddlia chondrophila*, cell division, peptidoglycan, sporulation

## Abstract

*Chlamydiales* species are obligate intracellular bacteria lacking a classical peptidoglycan sacculus but relying on peptidoglycan synthesis for cytokinesis. While septal peptidoglycan biosynthesis seems to be regulated by MreB actin and its membrane anchor RodZ rather than FtsZ tubulin in *Chlamydiales*, the mechanism of peptidoglycan remodeling is poorly understood. An amidase conserved in *Chlamydiales* is able to cleave peptide stems in peptidoglycan, but it is not clear how peptidoglycan glycan strands are cleaved since no classical lytic transglycosylase is encoded in chlamydial genomes. However, a protein containing a SpoIID domain, known to possess transglycosylase activity in Bacillus subtilis, is conserved in *Chlamydiales*. We show here that the SpoIID homologue of the *Chlamydia*-related pathogen Waddlia chondrophila is a septal peptidoglycan-binding protein. Moreover, we demonstrate that SpoIID acts as a lytic transglycosylase on peptidoglycan and as a muramidase on denuded glycan strands *in vitro*. As SpoIID-like proteins are widespread in nonsporulating bacteria, SpoIID might commonly be a septal peptidoglycan remodeling protein in bacteria, including obligate intracellular pathogens, and thus might represent a promising drug target.

## Abstract (precis)

*Chlamydiales* species are obligate intracellular bacteria and important human pathogens that have a minimal division machinery lacking the proteins that are essential for bacterial division in other species, such as FtsZ. Chlamydial division requires synthesis of peptidoglycan, which forms a ring at the division septum and is rapidly turned over. However, little is known of peptidoglycan degradation, because many peptidoglycan-degrading enzymes are not encoded by chlamydial genomes. Here we show that an homologue of SpoIID, a peptidoglycan-degrading enzyme involved in sporulation of bacteria such as Bacillus subtilis, is expressed in *Chlamydiales*, localizes at the division septum, and degrades peptidoglycan *in vitro*, indicating that SpoIID is not only involved in sporulation but also likely implicated in division of some bacteria.

## INTRODUCTION

Cell division is an essential step for proliferation of all bacteria that is executed after genome replication. Typically, the assembly of the division septum is organized by the bacterial tubulin homolog FtsZ, which polymerizes at the division septum and recruits important proteins involved in the remodeling of the peptidoglycan (PG), subsequently directing the invagination of the envelope (1). PG is a polymer of glycan strands composed of β-1,4-linked *N*-acetylglucosamine (GlcNAc) and N-acetylmuramic acid (MurNAc) disaccharide units that are cross-linked by peptide bridges. The composition of the peptide bridges can differ among different species but typically contains both d- and l-amino acids. PG synthesis begins in the cytoplasm and at the cytoplasmic membrane to form lipid II. Once lipid II is flipped to the extracytosolic space, the penicillin-binding proteins (PBPs) and/or the proteins of the shape, elongation, division and sporulation (SEDS) family execute the transglycosylation and transpeptidation reactions to generate glycan strands and peptide cross-links, respectively (2–4). PBP activity is required at the division septum and is inhibited by penicillin and other beta-lactam antibiotics that mimic parts of lipid II. Interestingly, few bacteria can remodel their PG at the division septum in the absence of FtsZ. Among these bacteria are *Chlamydiales* species (2).

*Chlamydiales* are Gram-negative (diderm) obligate intracellular bacteria, including many important human pathogens such as Chlamydia trachomatis and Chlamydia pneumoniae, which are classified in the Chlamydiaceae family. All members of the *Chlamydiales* order that are not part of the Chlamydiaceae family are commonly referred to as *Chlamydia*-related bacteria. They have been much less extensively studied but include putative emergent pathogens such as Waddlia chondrophila (for reviews, see references 2 and 5). *Chlamydiales* underwent a drastic genome reduction apparently driven by the adaptation of these bacteria to an intracellular parasitic lifestyle (6–8) but still retained all minimal determinants required for cell division and to synthesize and remodel PG. They are thus excellent models to elucidate the requirements for a minimal bacterial division machine. PG could be detected as a ring in dividing Chlamydiaceae (9, 10) and as a complete sacculus in some *Chlamydia*-related bacteria (11). The very small amount of PG detected in W. chondrophila argues against the present of a complete septum in this species (12), but corroborating evidence is lacking thus far. As *Chlamydiae* lack FtsZ, they appear to rely on the actin homologue MreB and its membrane anchor RodZ (13, 14) to regulate PG synthesis and remodeling in time and space (9, 10). However, the mechanism and the regulation of PG remodeling at the division septum in *Chlamydiae* are not fully understood. Amidases able to cleave peptide stems at the amide bond of MurNAc were described in Waddlia chondrophila and Chlamydia pneumoniae (15, 16), but chlamydial genomes do not code for homologues of classical lytic transglycosylases (17), raising the issue of how the glycan strand is cleaved. Interestingly, a gene coding for a protein containing a SpoIID-domain is conserved among the members of *Chlamydiales*. Since a SpoIID-domain containing protein of Bacillus subtilis was demonstrated previously to be a lytic transglycosylase involved in sporulation (18, 19), this activity might be conserved in *Chlamydiales*.

In this study, we showed that a protein containing a SpoIID domain (here called SpoIID^Wch^ [for “W. chondrophila SpoIID”] for clarity) is able to bind PG. SpoIID^Wch^ is septally localized and was shown to be capable of being immunoprecipitated together with RodZ. Furthermore, chlamydial SpoIID, similarly to its B. subtilis homologue, binds PG *in vitro* and digests denuded glycan chains (i.e., those with no peptide stem attached). However, in contrast to B. subtilis SpoIID (SpoIID^Bsu^), SpoIID^Wch^ seems to act as a lytic transglycosylase on Gram-negative PG *in vitro* and as a muramidase on denuded glycan chains, indicating that it might possess dual activities, depending on its substrate. Note that proteins containing a SpoIID domain are present in a large variety of both sporulating bacteria and nonsporulating bacteria, indicating that this domain may fulfill an ancestral role in PG remodeling and cell division, independently of sporulation and before branching of cyanobacteria, firmicutes, and proteobacteria, as already suggested previously by Morlot et al. (18).

## RESULTS

### Waddlia chondrophila peptidoglycan can be detected in small amounts.

**FIG 1:**
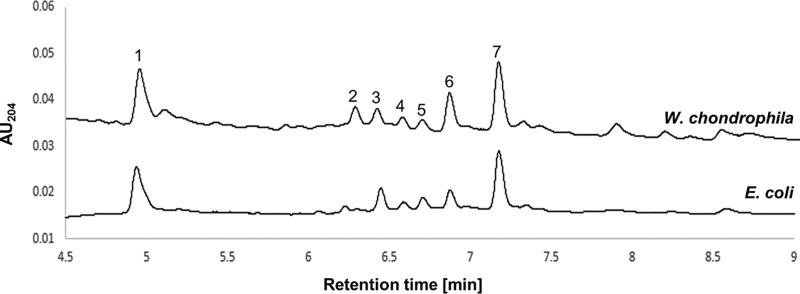
Detection of PG of Waddlia chondrophila by UPLC-QTOF-MS. Vero cells infected by W. chondrophila were lysed with a solution of phenol/ethanol/SDS. The insoluble fraction was then boiled in SDS and washed before analysis by UPLC-QTOF-MS. The numbered peaks were characterized with the help of their MS and MS/MS fragmentation patterns. Potential structures of the numbered peaks are depicted in Table 1 (see also Fig. S1). AU, absorbance unit.

In an earlier study, we showed that extracts from W. chondrophila were able to activate the Nod pathway of a reporter mammalian cell (12). However, the low concentrations of PG obtained were not sufficient to characterize its structure by ultraperformance liquid chromatography (UPLC) and subsequent mass spectrometry (MS). To improve PG extraction, we used 240 flasks (25 cm^2^) of W. chondrophila-infected Vero cells. Sufficient amounts of PG could be obtained and analyzed by the use of advanced and highly sensitive UPLC-QTOF-MS (UPLC-quadrupole time of flight-MS) (Fig. 1). The chemical structures corresponding to the W. chondrophila PG peaks were determined using their MS and tandem MS (MS/MS) fragmentation patterns (Table 1; see also Fig. S1A in the supplemental material). These peaks (numbered 1 to 7) were assigned to molecular weights corresponding to PG subunits. For example, peak 1 corresponds to a PG monomer composed of a disaccharide tetrapeptide, GlcNAc-MurNAc-l-Ala-d-Glu-mDAP-d-Ala (M4; Fig. S1A), and peak 7 corresponds to two disaccharides linked together by eight amino acids (aa) (D44; Fig. S1A). All seven peaks detected and analyzed (Table 1; see also Fig. S1A) correspond to PG subunits similar to those found in classical Gram-negative PG (e.g., Escherichia coli) and in C. trachomatis PG (20). However, the number of ld cross-links is apparently higher in W. chondrophila than in model Gram-negative bacteria such as E. coli (Fig. 1, peaks 2 and 6).

**TABLE 1:** UPLC-QTOF-MS analysis of muropeptides from W. chondrophila PG (Fig. 1)

Peak no.	Retention time (min)	% content	[M + H]^+^ (calculated)	[M + H]^+^ (observed)	Mass difference (cal/obs)^*a*^	Muropeptide
1	4.94	23.31	942.4147			M4^*b*^
2	6.24	5.82	1,780.7591	1,780.7558	0.0033	D33G
3	6.37	4.88	1,723.7376	1,723.7349	0.0027	D33
4	6.53	3.07	1,851.7962	1,851.8025	0.0063	D43G/D34G
5	6.65	3.6	1,794.7747	1,794.7693	0.0054	D43
6	6.81	13.36	1,794.7747	1,794.7741	0.0006	D34
7	7.11	25.79	1,865.8118	1,865.7996	0.0122	D44

a cal/obs, calculated/observed.

b The identity of M4 was established by retention time matching with an E. coli PG reference (MS previously characterized) under the same separation conditions.

10.1128/mBio.01128-19.2 FIG S1 (A) Structures of the muropeptides listed in Table 1. (B) Phylogeny of SpoIID orthologs in the PVC (*Planctomycetes*, *Verrucomicrobia*, and *Chlamydiae*) superphylum. Alignments of the orthologs of SpoIID present in the PVC superphylum were performed as described in Text S1 and used to build a phylogeny. Localization of the SpoIID domain (PF08486) on the protein is depicted on the right for each ortholog. Locus tags of SpoIID orthologs of cyanobacteria and B. subtilis used in this study are highlighted in red. Download FIG S1, PDF file, 0.2 MB. Copyright © 2019 Jacquier et al. 2019 Jacquier et al. This content is distributed under the terms of the Creative Commons Attribution 4.0 International license.

10.1128/mBio.01128-19.1 TEXT S1 Extensive description of the materials and methods used in this article, including strains and growth conditions, quantitative PCR, protein and peptidoglycan isolations, production of antibodies, and bioinformatics analyses. Download Text S1, DOCX file, 0.03 MB. Copyright © 2019 Jacquier et al. 2019 Jacquier et al. This content is distributed under the terms of the Creative Commons Attribution 4.0 International license.

### Proteins binding to peptidoglycan and interacting with division machinery might be important actors in chlamydial division.

Proteins binding to chlamydial PG might have an important role in the PG biosynthesis/degradation cycle. We recently used mass spectrometry to identify the proteins of W. chondrophila that bind to PG (12). We also recently showed that RodZ, a regulator of the actin homologue MreB, is an inner membrane protein that localizes at the division septum at early stages of W. chondrophila division (14). We thus hypothesized that proteins interacting both with PG and with the divisome would be candidate regulators of chlamydial division that might coordinate PG biosynthesis/remodeling/degradation at the division septum and divisome formation. Since we identified RodZ as an early divisome component (14), we performed a coimmunoprecipitation with anti-RodZ antibodies on infected and noninfected cell lysates, followed by MS-based proteomics, in order to identify uncharacterized divisome components. Candidates showing enrichment in infected samples compared to the noninfected control, with the exclusion of the usual contaminants such as ribosomal proteins, were selected (see Table S1 in the supplemental material). By comparing this list with a list of proteins of W. chondrophila binding to PG, we identified Wcw_0967 (WCW_RS04685) as a potential candidate interacting with both RodZ and PG.

10.1128/mBio.01128-19.9 TABLE S1 Peptides detected by mass spectrometry after coimmunoprecipitation using an anti-RodZ antibody. Download Table S1, XLSX file, 0.01 MB. Copyright © 2019 Jacquier et al. 2019 Jacquier et al. This content is distributed under the terms of the Creative Commons Attribution 4.0 International license.

**FIG 2:**
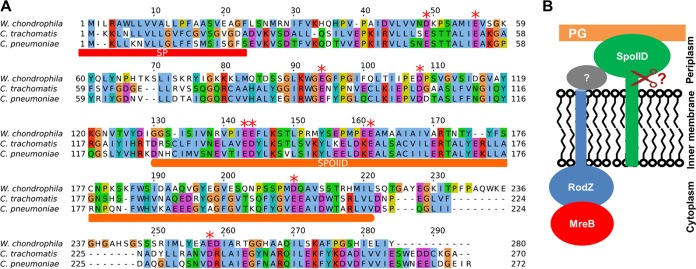
SpoIID is conserved among *Chlamydiae* and is predicted to localize to the periplasm. (A) Alignment of SpoIID homologues of W. chondrophila, C. trachomatis, and C. pneumoniae. SP, signal peptide; SpoIID, predicted SpoIID domain. Red stars indicate conserved glutamic acid/aspartic acid residues. Colored amino acids indicate conserved properties using the following Clustal X color scheme: blue, hydrophobic; red, positively charged; magenta, negatively charged; green, polar; pink, cysteines; orange, glycines; yellow, prolines; cyan, aromatic. (B) Working model showing the potential periplasmic localization of chlamydial SpoIID and its suggested interactions with PG and RodZ. The interactions with RodZ might be indirect and might occur through the activity of an undetermined divisome component (gray protein with a question mark). The scissor indicates a potential cleavage of the signal sequence located in the inner membrane.

The *wcw_0967* gene is conserved among *Chlamydiales*, encoding a protein with an N-terminal signal sequence that might be cleaved (Fig. 2A; see also Fig. S1B). Moreover, wcw_0967 contains a domain found in the stage II sporulation protein (SpoIID) of Bacillus subtilis. We thus renamed Wcw_0967 “SpoIID^Wch^.” SpoIID of B. subtilis (SpoIID^Bsu^) was shown to bind PG and drive membrane movement during B. subtilis sporulation, simultaneously degrading PG via its lytic transglycosylase activity (18). We were thus interested to investigate the potential role of the chlamydial homologues of SpoIID in the division process. We hypothesized that chlamydial SpoIID might link together PG biosynthesis/remodeling and divisome formation through interactions with the divisome (either directly with RodZ or through other divisome components) and PG (Fig. 2B). Moreover, chlamydial SpoIIDs might have a conserved lytic transglycosylase activity and might thus directly degrade septal PG.

### SpoIID^Wch^ is expressed early during the chlamydial developmental cycle and partially localizes to the division septum.

**FIG 3:**
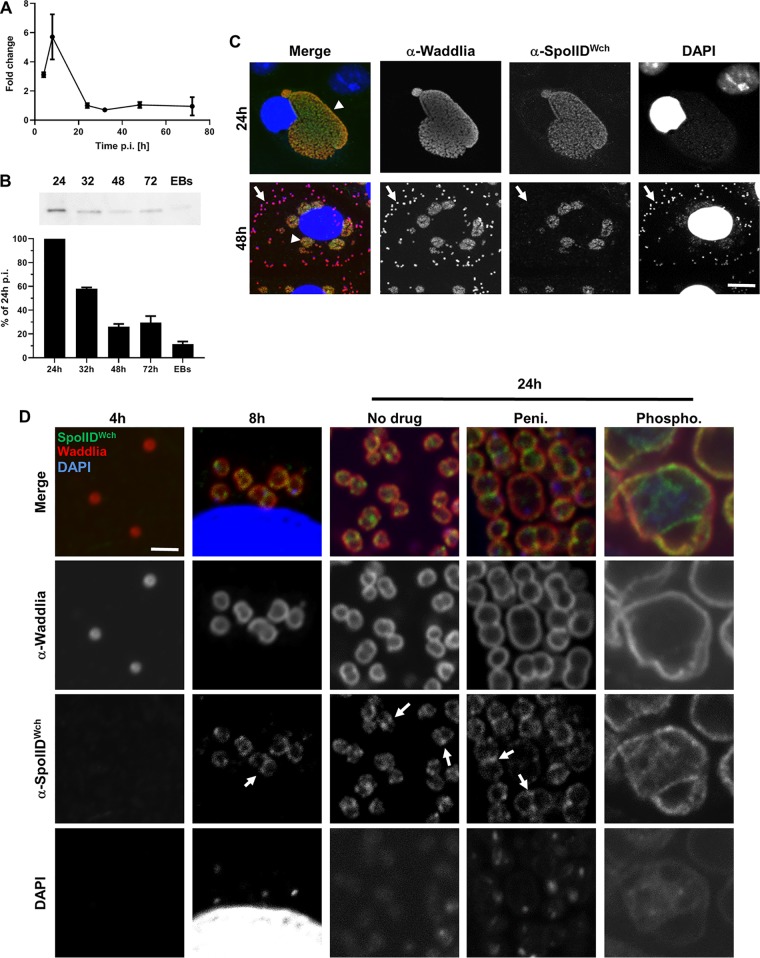
SpoIID^Wch^ is expressed early during the infection cycle and localizes at the division septum in RBs. (A) Measurement of transcription levels of *spoIID^Wch^* by qRT-PCR performed on total RNA extracted from Vero cells infected with W. chondrophila at the indicated time points. Transcripts levels are presented as fold changes compared to 16S rRNA results. (B) Measurement of protein levels by Western blotting at the indicated time points. Equivalent numbers of bacteria (quantified by qPCR) were loaded for each time point. (C) Immunofluorescence of Vero cells infected with W. chondrophila at the indicated time points (red, anti-*Waddlia* antibody; blue, DAPI; green, anti-SpoIID^Wch^ antibody). The arrowhead indicates an example of an RB expressing SpoIID^Wch^, and the arrows indicate EBs in which SpoIID^Wch^ was not detectable. Bar, 40 μm. (D) Immunofluorescence at different times p.i. under conditions similar to those described for panel C. Infected cells were treated or not with penicillin or phosphomycin 2 h p.i. Arrows show localization of SpoIID^Wch^ at the division septum. Scale bar 1 μm.

In order to investigate the potential role of SpoIID^Wch^ in division, we first measured the expression profile of *spoIID^Wch^* during infection of mammalian cells by W. chondrophila. We were able to detect mRNA by reverse transcription-quantitative PCR (qRT-PCR) at as early a time as 4 h postinfection (p.i.), with a peak 8 h p.i. (Fig. 3A). This expression pattern is consistent with a role of SpoIID^Wch^ in division, since it shows expression levels similar to what was observed previously for genes coding for members of the chlamydial divisome such as *mreB*, *rodZ*, and *pal* (12, 14). We then raised antibodies against SpoIID^Wch^ that specifically recognized a protein of the predicted size in samples taken at 32 and 48 h p.i. by immunoblotting (Fig. S4D). In order to normalize the quantity of protein and mRNA to the number of bacteria, quantitative PCR (qPCR) targeting 16S DNA (Fig. S4A), qRT-PCRs targeting 16S RNA (Fig. S4B) and SpoIID^Wch^ mRNA (Fig. S4C), and Western blotting were performed in parallel. qPCR was used to load protein samples with equivalent numbers of bacteria and to detect SpoIID^Wch^ by Western blotting (Fig. 3B). This was possible only from 24 h p.i. on, since the number of bacteria was too low at earlier time points. The quantity of proteins was found to be decreasing at late time points, indicating that SpoIID^Wch^ was present mainly in intracellular replicating reticulate bodies (RBs). SpoIID^Wch^ could be detected in RBs at both 24 and 48 h p.i. (Fig. 3C, arrowheads), but elementary bodies (EBs), the extracellular infective forms, which are recognizable by their strong DAPI (4′,6-diamidino-2-phenylindole) staining due to DNA condensation, were not labeled by these antibodies (Fig. 3C, arrows). We cannot exclude the possibility that the lack of detection of SpoIID^Wch^ by immunofluorescence in EBs was due to poor permeability of EBs. However, the low level of detection of SpoIID^Wch^ in purified EBs by immunoblotting (Fig. 3B) is a good indication that this protein is not strongly present in EBs, since many proteins were able to be detected earlier in EBs by Western blotting as shown previously (21). Interestingly, the antigen could be detected by immunofluorescence at as early a time point as 8 h p.i. (Fig. 3D), which is consistent with the early RNA expression (Fig. 3A).

10.1128/mBio.01128-19.5 FIG S4 (A) Detection of 16S DNA at the indicated time points in Vero cells infected with W. chondrophila by qPCR. (B and C) Quantification of 16S RNA and *spoIID^Wch^* mRNA by qRT-PCR. Results were normalized to the number of bacteria using the qPCR data from panel A. (D) Western blotting using anti-SpoIID^Wch^ on samples harvested at the indicated time points. Equivalent volumes of sample were loaded on all lines. (E and F) PG sacculi of E. coli or V. cholerae were incubated with SpoIID^Wch^ or not incubated with SpoIID^Wch^ (control) overnight at 37°C. The resulting soluble moieties were analyzed by UPLC-QTOF. The identity of the peaks was determined by MS/MS. M4N, anhydro glycan with 4 peptides; ?, uncharacterized PG moiety. (G and H) MS/MS spectra of selected peaks from the data shown in Fig. 5B. MS/MS analysis revealed anhydro analogues. Download FIG S4, PDF file, 0.1 MB. Copyright © 2019 Jacquier et al. 2019 Jacquier et al. This content is distributed under the terms of the Creative Commons Attribution 4.0 International license.

In a further step, the subcellular localization of SpoIID in W. chondrophila was investigated using higher-resolution fluorescence microscopy. We observed partial septal localization of SpoIID^Wch^ in dividing RBs at 24 h p.i. (Fig. 3D, arrows). This septal localization was confirmed by quantification of fluorescence signal (Fig. S2A). However, we observed accumulation of SpoIID^Wch^ not only at the division septum but also in some foci. Treatment of W. chondrophila with penicillin induces aberrant bodies in which septation is stalled and divisome components such as RodZ are accumulating at the septum (14). SpoIID^Wch^ localization at the division septum was not affected by penicillin treatment (Fig. 3D, arrows; see also Fig. S2B). Moreover, the septal localization of SpoIID^Wch^ was dependent on PG biosynthesis, since treatment with phosphomycin, which inhibits a lipid II biosynthesis step in the cytoplasm, completely dispersed SpoIID^Wch^ from midcell (Fig. 3D; see also Fig. S3). Thus, SpoIID requires PG precursors to localize to the septum, similarly to what was observed previously for RodZ (14).

10.1128/mBio.01128-19.3 FIG S2 Quantification of the localization of SpoIID^Wch^ in the untreated (A) and penicillin-treated (B) cells described in the Fig. 3D legend. Three cells were selected, and fluorescence was measured along the septum (a), along the sides of the dividing bacterium (b and c), and along the center of the dividing bacteria (d). Quantification of SpoIID^Wch^ (green), and anti-W. chondrophila polyclonal antibody staining of the outer membrane (red), and DAPI staining (blue) were performed by the use of ImageJ. Download FIG S2, PDF file, 0.1 MB. Copyright © 2019 Jacquier et al. 2019 Jacquier et al. This content is distributed under the terms of the Creative Commons Attribution 4.0 International license.

10.1128/mBio.01128-19.4 FIG S3 Quantification of the localization of SpoIID^Wch^ in cells treated with phosphomycin as described for Fig. 3D (penicillin panel). Three cells were selected, and fluorescence was measured along the septum (a), along the sides of the dividing bacterium (b and c), and along the center of the dividing bacteria (d). Quantifications of SpoIID^Wch^ (green), anti-W. chondrophila polyclonal antibody staining the outer membrane (red), and DAPI staining (blue) were performed by the use of ImageJ. Download FIG S3, PDF file, 0.1 MB. Copyright © 2019 Jacquier et al. 2019 Jacquier et al. This content is distributed under the terms of the Creative Commons Attribution 4.0 International license.

### SpoIID^Wch^ binds to PG *in vitro*.

**FIG 4:**
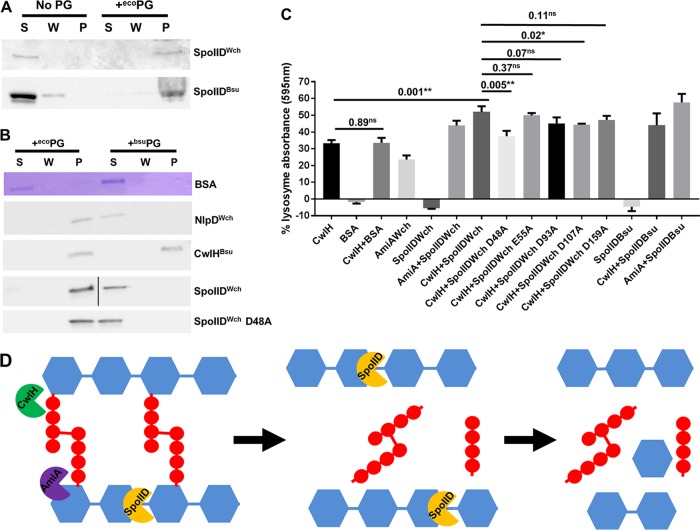
SpoIID binds to E. coli PG and digests naked glycan strands of PG. (A) Recombinant SpoIID^Wch^ and SpoIID^Bsu^ lacking N-terminal transmembrane domain were purified. The protein was soluble in the absence of PG but cosedimented with E. coli PG when the sample was incubated for 30 min on ice with PG and was then centrifuged at 20,000 × *g* for 30 min. Proteins were then separated by SDS-PAGE and detected by Western blotting. S, supernatant; W, wash; P, pellet. (B) PGs of E. coli and B. subtilis were incubated with the indicated recombinant proteins for 30 min on ice. The suspension was then centrifuged at 20,000 × *g* for 30 min and washed once. Supernatant (S), wash (W), and pellet (P) fractions were then analyzed by SDS-PAGE followed or not followed by Western blotting, when antibodies were available. (C) RBB-labeled E. coli PG was incubated with the indicated recombinant proteins for 30 min at 37°C. The suspensions were then sedimented by centrifugation at 20,000 × *g* for 30 min. Absorption of the supernatant was measured in order to quantify the amounts of soluble PG moieties that were released. The data represent the means of results from three independent experiments, expressed in percentages of the positive-control lysozyme levels. Error bars show standard deviations, and numbers represent *P* values from unpaired *t* tests. Stars indicate *P* values lower than 0.005 (**) and lower than 0.05 (*); ns, not significant. (D) Model depicting the sequential roles of CwlH and/or AmiA in digesting the peptide bounds (in red) and of SpoIID, which was able to digest the naked glycan strands (in blue), leading to the release of soluble PG moieties.

The B. subtilis homologue of SpoIID was shown to bind to PG and to digest naked glycan strands of PG (18). We thus decided to investigate if these properties are conserved for SpoIID^Wch^. We could purify soluble versions of SpoIID^Wch^ and SpoIID^Bsu^ that lacked their N-terminal signal peptides (Fig. 4A). Fittingly, after coincubation of SpoIID^Wch^ or SpoIID^Bsu^ with purified PG sacculi from the Gram-negative E. coli, we observed cosedimentation of the proteins with the PG, indicating that both SpoIID^Wch^ and SpoIID^Bsu^ are indeed able to directly bind PG (Fig. 4A). Interestingly, SpoIID^Wch^ was unable to bind PG from the Gram-positive bacterium B. subtilis (Fig. 4B). Another W. chondrophila protein, NlpD, which was shown to directly bind to PG (15), also bound only to E. coli PG. In contrast, the B. subtilis amidase CwlH, which was shown previously to produce naked glycan chains (22), was able to bind to both Gram-positive and Gram-negative sacculi (Fig. 4B).

### SpoIID^Wch^ degrades naked glycan strands *in vitro*.

We then investigated the activity of SpoIID^Wch^ on PG *in vitro* using a PG release assay. This assay helps efforts designed to estimate the activity of a protein on PG by quantifying the release of a dye from PG stained with Remazol brilliant blue (RBB) (18). Incubation of labeled PG with bovine serum albumin (BSA) as a control protein did not result in any detectable release of dye in the supernatant. Interestingly, addition of SpoIID^Wch^ alone did not induce any visible dye release in this assay, similarly to what was observed for SpoIID^Bsu^ (18) (Fig. 4C). Indeed, SpoIID^Bsu^ was shown to digest only naked glycan strands of PG, after removal of the stem peptides (18). To determine whether SpoIID^Wch^ can only digest naked glycan strands, we used the B. subtilis amidase CwlH^Bsu^ in an assay similar to what was performed with SpoIID^Bsu^ (22). RBB-labeled E. coli PG was thus incubated simultaneously with CwlH^Bsu^ and SpoIID^Wch^ during 30 min. This coincubation caused a significant 1.5× increase in dye release compared to CwlH^Bsu^ alone (Fig. 4C). This effect was specific to SpoIID and was not mimicked by the addition of an unrelated protein, since coincubation of PG with BSA and CwlH^Bsu^ did not cause an increase in the level of dye release. Interestingly, SpoIID^Wch^ showed a level of activity similar to that seen with SpoIID^Bsu^ (Fig. 4C), consistent with the notion that SpoIID^Wch^ can digest naked glycan strands (Fig. 4D). Interestingly, AmiA^Wch^, a chlamydial amidase (15, 16), showed activity with respect to PG that was similar to that seen with CwlH^Bsu^ and allowed digestion of PG by SpoIID^Wch^ (Fig. 4D), indicating that SpoIID^Wch^ might have access to naked PG *in vivo*.

### SpoIID^Wch^ acts as a lytic transglycosylase on PG *in vitro*.

**FIG 5:**
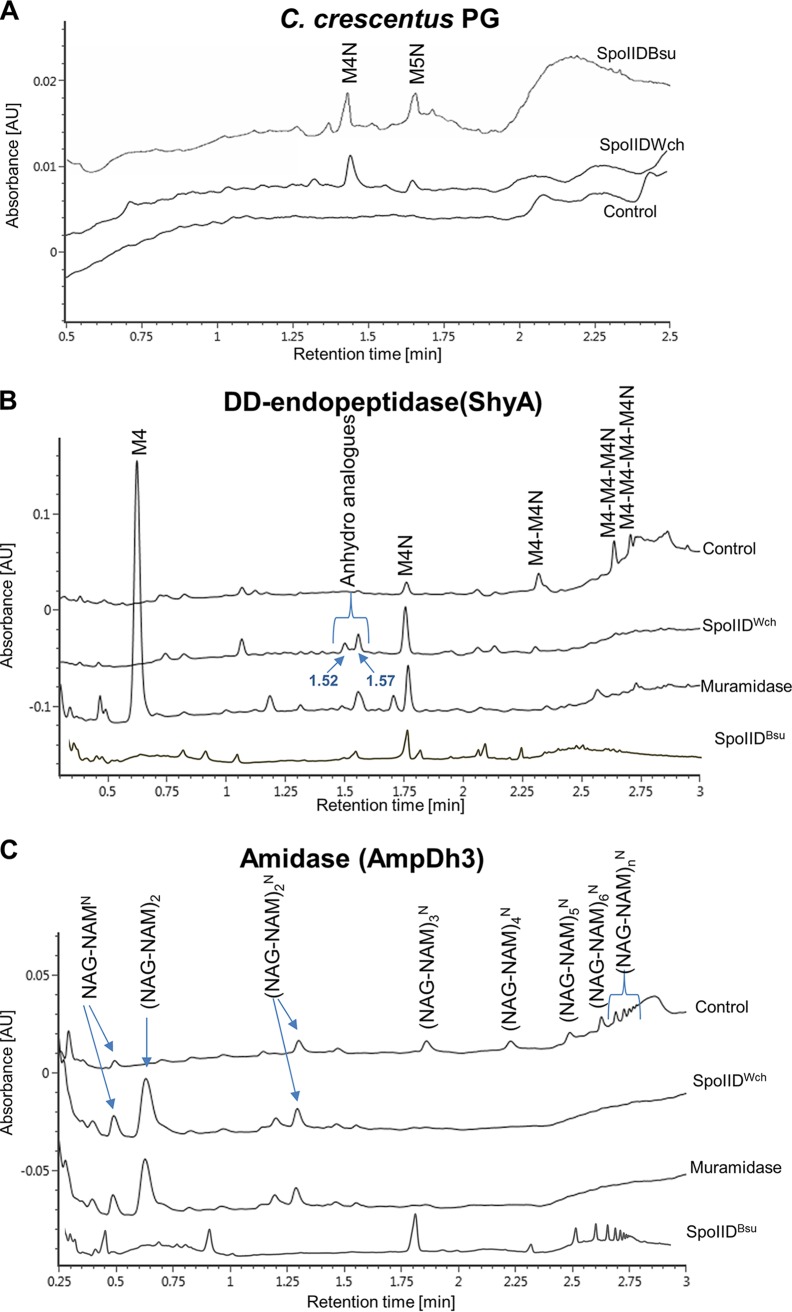
SpoIID^Wch^ shows a lytic transglycosylase activity on PG sacculi and a muramidase activity on denuded glycans. (A) PG sacculi of Caulobacter crescentus were incubated with SpoIID^Wch^ or with SpoIID^Bsu^ or were incubated in the absence of enzyme (control) overnight at 37°C. The resulting soluble moieties were analyzed by UPLC-QTOF. The identities of the peaks were determined by MS/MS. M4N, GlcNac-anhydroMurNac with 4 amino acids; M5N, GlcNac-anhydroMurNac with 5 amino acids. (B) PG sacculi of Vibrio cholerae were digested with ShyA, a d,d-endopeptidase, and then incubated with SpoIID^Wch^ or with SpoIID^Bsu^ or with a muramidase or without an enzyme (control). The resulting soluble moieties were analyzed as described for panel A. (C) PG sacculi of V. cholerae were digested with the amidase AmpDh3 and then incubated overnight with SpoIID^Wch^ or with SpoIID^Bsu^ or with a muramidase or without an enzyme (control). Soluble moieties were analyzed similarly to the manner described for panel A. NAG, *N*-acetylglucosamine; NAM, N-acetyl-muramic acid. Superscript “N,” anhydro.

To further investigate the activity of SpoIID^Wch^ on PG, purified SpoIID^Wch^ was incubated overnight at 37°C with PG of different bacterial species. Digested PG was then analyzed by UPLC-QTOF. Interestingly, SpoIID^Wch^ showed low levels of activity on PG of E. coli, Vibrio cholerae, and Caulobacter crescentus, causing the release of low levels of anhydro moieties (see M4N [GlcNAc-anhydroMurNAc-l-Ala-d-Glu-mDAP-d-Ala] and M5N [GlcNac-anhydroMurNac with 5 amino acids] data in Fig. 5A; see also M4N data in Fig. S4E and F). Interestingly, SpoIID^Wch^ caused release of M5N only from PG of C. crescentus, indicating that this PG might have slight structural changes that could expose these moieties to the enzyme. A similar level of activity was observed when SpoIID^Bsu^ was incubated with PG of C. crescentus (Fig. 5A). However, we cannot exclude the possibility that SpoIID^Wch^ has dual activities on PG of E. coli and V. cholerae, since we noted the appearance of a secondary peak, whose structure cannot be determined at this point (labeled with question marks in Fig. S4E and F). Taken together, these results indicate that SpoIID^Wch^ has low but detectable lytic transglycosylase activity on PG sacculi, since overnight incubation was required for detectable release. This activity was confirmed by incubation of SpoIID^Wch^ with PG from V. cholerae pretreated with ShyA, a d,d-endopeptidase of V. cholerae (23). Under these conditions, SpoIID^Wch^ was able to release the GlcNAc-anhydroMurNAc-l-Ala-d-Glu-mDAP-d-Ala (M4N) along with other anhydro-muropeptides (anhydro analogues; 1.52 and 1.57 min of retention time) (Fig. 5B; see also Fig. S4G and H). Similar peaks were detected when SpoIID^Bsu^ was used (Fig. 5B). Finally, incubation of SpoIID^Wch^ with denuded glycan chains produced by digestion of PG from V. cholerae with the amidase AmpDh3 from Pseudomonas aeruginosa induced the release of a majority of nonanhydro moieties, similarly to treatment with muramidase, which cleaves between MurNAc and GlcNAc [(NAG-NAM)_2_; Fig. 5C]. This is in contrast with the activity of SpoIID^Bsu^, which did not cause accumulation of nonanhydro moieties (Fig. 5C). Taken together, these results indicate that SpoIID^Wch^ might be a bifunctional lytic transglycosylase/muramidase and that its activity, which is different from that of SpoIID^Bsu^, might depend on its substrate.

### An aspartic residue at position 48 is important for SpoIID^Wch^ activity.

A glutamic acid residue (E88) was shown to be essential for the activity of SpoIID^Bsu^ (18). We wondered if a similar residue would be conserved and essential for the activity of SpoIID^Wch^. We selected conserved glutamic acid and/or aspartic acid residues (since the two have similar properties) in SpoIID of W. chondrophila, C. trachomatis, and C. pneumoniae (Fig. 2A, red stars). We designed 6 different point mutants of SpoIID^Wch^: D48A, E55A, E93A, D107A, EE141-142AA, and E159A. We were able to express soluble versions of all mutants except the double mutant EE141-142AA, which was insoluble, possibly because of improper folding caused by the introduced mutations. However, the five other mutants were soluble and able to bind PG to the same extent as the wild-type version (Fig. 4B; see also Fig. S5A to C). We investigated their activity in a PG-release assay and observed that all showed activity on PG that was not significantly different from that seen with the wild-type strain, with the exception of the D48A mutant, which showed reduced activity (Fig. 4C). Interestingly, this residue is conserved either as a glutamic acid or as an aspartic acid among all sequenced *Chlamydiales* spp. (Fig. S1B).

**FIG 6:**
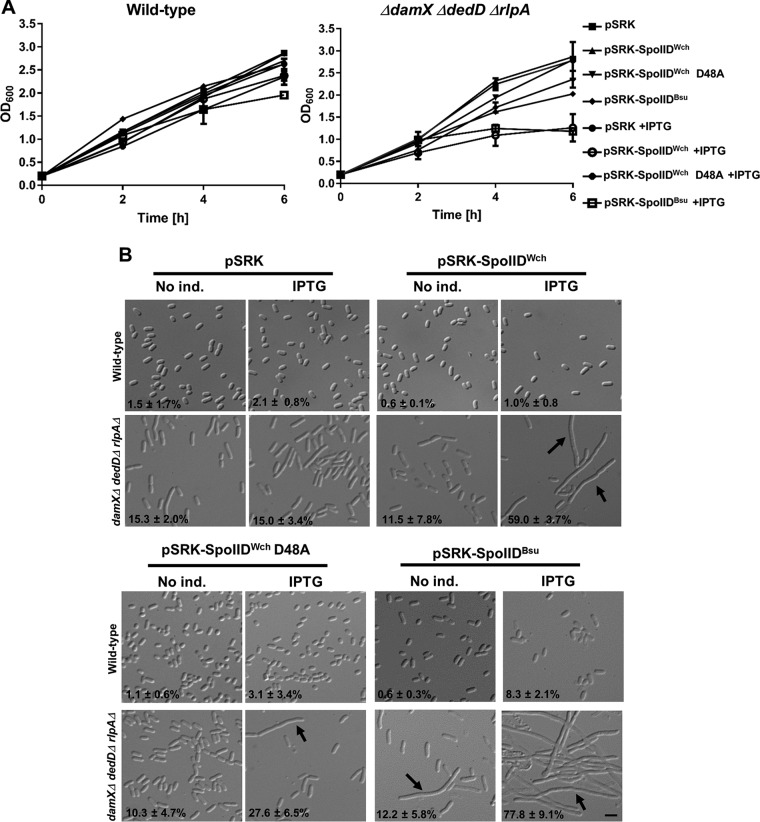
Overexpression of SpoIID^Wch^ in E. coli induces growth defect in a manner similar to that seen with SpoIID^Bsu^ only in the absence of three SPOR proteins. (A) Plasmids allowing expression of an untagged version of SpoIID^Wch^ or a mutant version of SpoIID^Wch^ D48A or SpoIID^Bsu^ fused with the 21-aa N-terminal signal sequence of the periplasmic protein TrbC from E. coli or the empty corresponding plasmid (pSRK) were transformed in the indicated strains. Overnight cultures were diluted to an OD_600_ of 0.2/ml and grown for 1 h, and IPTG was added (IPTG) or not (No ind.). The optical density of the cultures was measured at the indicated time points for two independent experiments. Error bars represent standard deviations of results from two independent experiments. (B) Liquid cultures treated as described for panel A were harvested 6 h after dilution and observed by microscopy. Arrows highlight elongated bacteria. Elongated (>5-μm-long) bacteria were counted using ImageJ software, and percentages of elongated bacteria were calculated (bar, 4 μm).

10.1128/mBio.01128-19.6 FIG S5 (A and B) E. coli expressing the indicated point mutants or the wild-type version of SpoIID^Wch^ was lysed, and the lysate was centrifuged at 20,000 × *g* for 10 min. Equivalent amounts of pellet and supernatant were separated by SDS-PAGE and subjected to Coomassie staining. (C) PG of E. coli was incubated with the indicated purified recombinant proteins for 30 min on ice. The suspension was then centrifuged at 20,000 × *g* for 30 min and washed once. Supernatant (S), wash (W), and pellet (P) fractions were then analyzed by Western blotting. (D) Wild-type and mutant versions of SpoIID^Wch^ are equally expressed in E. coli. The indicated strains were treated as described for Fig. 6A. Equivalent amounts of bacteria (OD_600_ of 1) were harvested at 6 h with or without addition of IPTG and resuspended in Laemmli buffer. The protein was then detected by Western blotting using polyclonal mouse anti-SpoIID^Wch^. Bands marked with stars are nonspecific due to background immunity of mice against E. coli. (E) Measurement of CFU per OD_600_ upon overexpression of SpoIIDs. The indicated strains were grown, and production of SpoIIDs was induced or not induced by the addition of IPTG. After 6 h of induction, OD_600_ levels were measured, serial dilutions were spread on LB plates, and colonies were counted. Download FIG S5, PDF file, 0.2 MB. Copyright © 2019 Jacquier et al. 2019 Jacquier et al. This content is distributed under the terms of the Creative Commons Attribution 4.0 International license.

### Overexpression of SpoIID^Wch^ in E. coli caused a growth defect in the absence of three SPOR proteins.

In order to test the activity of SpoIID^Wch^ *in vivo*, we overexpressed this protein in E. coli. Overexpression of SpoIID^Wch^ in a wild-type E. coli strain, akin to overexpression of SpoIID^Bsu^, did not cause any growth defect or filamentation (Fig. 6). We hypothesized that, in the wild-type strain, denuded PG could be protected from SpoIID activity, perhaps through the action of other PG-binding proteins and/or substrate limitation. Three SPOR domain-containing proteins, DamX, DedD, and RlpA, were recently described as being septally localized in E. coli (24) and as binding denuded PG (25). We thus overexpressed SpoIID^Wch^ and SpoIID^Bsu^ from a pSRK plasmid containing a promoter inducible by IPTG (isopropyl-β-d-thiogalactopyranoside) in single mutants Δ*damX*, Δ*dedD*, and Δ*rlpA* and in a triple mutant. IPTG-induced overexpression of SpoIIDs caused a growth defect in the triple mutant only (Fig. 6A), which is consistent with our hypothesis that the SPOR proteins might protect PG from the activity of SpoIIDs. Moreover, overexpression of SpoIIDs in the triple mutant induced a strong filamentation phenotype (Fig. 6B), indicating that the activity of SpoIIDs apparently blocks E. coli division. Interestingly, the viability of the bacteria was apparently not affected by overexpression of SpoIID^Wch^ but was partially affected by overexpression of SpoIID^Bsu^, as measured by CFU counting (Fig. S5E). Overexpression of the point mutant SpoIID^Wch^ D48A, which reached levels of proteins similar to those seen with the wild-type version (Fig. S5D), induced a lesser growth defect and a lesser filamentation phenotype (32% elongated bacteria compared to 56% for the wild-type version), further confirming its important role in the activity of SpoIID^Wch^ (Fig. 6). Taken together, these results are favor the idea of conservation of activity between SpoIIDs of different species. However, we cannot exclude the possibility that overexpression of SpoIIDs in E. coli causes growth defect independently of the activity of SpoIIDs on PG. Nevertheless, the phenotypes showing lower levels of growth and filamentation observed with the D48A mutant, which is expressed at a level similar to that at which its wild-type counterpart is expressed, indicate that these phenotypes depend on SpoIID activity.

### Conservation of proteins containing SpoIID domains in nonsporulating bacteria.

**FIG 7:**
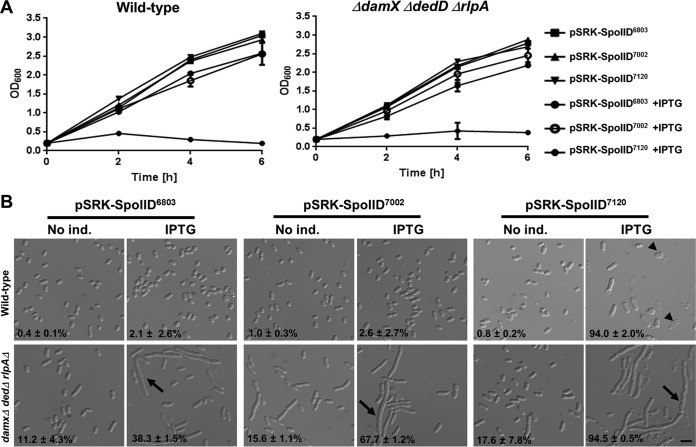
Overexpression of cyanobacterial SpoIID homologues causes growth defect in E. coli. SpoIID homologues of *Cyanobacteria* species *Synechocystis* sp. strain PCC6803, *Synechococcus* sp. strain PCC7002, and *Nostoc* sp. strain PCC7120 were cloned in a pSRK vector under the control of an IPTG-inducible promoter. (A) The effects of the expression of the indicated genes on growth of the strains mentioned above were determined by measurement of optical density (OD_600_) at different time points after IPTG induction. (B) Effects of the overexpression of these genes on the morphology of the indicated strains were investigated by microscopy, and elongated bacteria were counted as described for Fig. 6. Arrowheads indicate lysed cells, and arrows indicate elongated bacteria (bar, 4 μm).

SpoIID activity was investigated mainly in bacteria undergoing sporulation. However, the SpoIID domain is conserved in many nonsporulating bacteria (18). This is consistent with the fact that SpoIID domain-containing proteins can also be found in bacterial clades that have apparently lost other sporulation proteins such as the sporulation regulator Spo0A, the sporulation-specific small acid-soluble protein SspA, and the enzyme involved in the synthesis of dipicolinate and the main component of bacterial spores, DpaA (Fig. S7). It was especially interesting to observe that SpoIID was conserved in a large majority of cyanobacteria, which are completely devoid of sporulation markers. We expressed cyanobacterial SpoIID from three model organisms of cyanobacteria, namely, *Synechocystis* sp. strain PCC6803, *Synechococcus* sp. strain PCC7002, and *Nostoc* sp. strain PCC7120, in a wild-type E. coli strain and in the Δ*damX* Δ*dedD* Δ*rlpA* triple mutant. Overexpression of *Synechocystis* and *Synechococcus* SpoIIDs caused no growth defect in the wild-type strain and only a moderate filamentation in the triple mutant (Fig. 7). In contrast, *Nostoc* SpoIID induced a strong growth defect in both the wild-type strain and the triple mutant (Fig. 7A). Moreover, *Nostoc* SpoIID overexpression caused lysis of the wild-type strain and filamentation of the triple mutant (Fig. 7B). These results indicate that cyanobacterial SpoIID seems to have an effect on the E. coli cell wall similar to the effect of SpoIID^Wch^, although the extents of the effects differ.

10.1128/mBio.01128-19.8 FIG S7 The SpoIID domain is more widespread in the bacterial kingdom than sporulation markers SpoA, SspA, and DpaA. Conservation of the SpoIID domain (green, PF08486.9), SpoOA domain (pink, PF08769.10), SspA domain (blue, PF00269.19), and DpaA domain (orange, PF16924.4) in a selection of bacterial, archeal, and eukaryotic genomes is shown here. The length of the colored bar represents the percentage of species among the indicated order coding for a SpoIID homologue. Download FIG S7, PDF file, 0.2 MB. Copyright © 2019 Jacquier et al. 2019 Jacquier et al. This content is distributed under the terms of the Creative Commons Attribution 4.0 International license.

## DISCUSSION

Proteins containing a SpoIID domain are well conserved in many diverse bacterial species. SpoIID of B. subtilis has been well studied and was shown previously to be a lytic transglycosylase required for PG remodeling during sporulation of this bacterium (18). Interestingly, the chlamydial SpoIID homologue protein might provide the transglycosylase activity required for rapid and complete PG degradation, similarly to its homologue in B. subtilis. Indeed, the SpoIID protein was detected as a PG-binding protein in a screen that we performed to investigate W. chondrophila proteins cosedimenting with PG (data not shown). Moreover, SpoIID could also be coimmunoprecipitated with the MreB membrane anchor RodZ. Chlamydial SpoIIDs show only a low level of amino acid identity with SpoIID^Bsu^ (19.9% for W. chondrophila and 19.0% for C. trachomatis). Moreover, chlamydial SpoIIDs are quite divergent from each other, with a low identity level of 23.74% between SpoIID^Wch^ and SpoIID^Ctr^ (percentages of identity are calculated from the alignments presented in Fig. S1B in the supplemental material). However, the SpoIID domain is conserved, as are several glutamic acid and aspartic acid residues (Fig. 2, red stars). This is of interest since the activity of SpoIID^Bsu^ requires a glutamic acid in the active site (22). We were able to show that SpoIID^Wch^, despite its low level of similarity to SpoIID^Bsu^, has similar activity *in vitro* with respect to PG. This level of activity was lowered when an aspartic acid present at position 48 was mutated (Fig. 4C). Interestingly, E. coli lacking SPOR proteins was sensitive to overexpression of both SpoIID^Wch^ and SpoIID^Bsu^. This is consistent with a potential important role of SpoIID as a PG-binding protein. However, overexpression of SpoIID^Wch^ in C. trachomatis had no clear effect on proliferation or on bacterial size (Fig. S6). Bacterial size measurements were slightly lower upon overexpression of SpoIID^Wch^, but this could be explained by the indirect effect of protein overexpression on the chlamydial mechanism. Taken together, these results indicate that overexpression of SpoIID^Wch^ might not be sufficient to disturb C. trachomatis proliferation and that PG-modifying protein levels might be tightly regulated in *Chlamydiales*, or PG might be protected by other PG-binding proteins.

10.1128/mBio.01128-19.7 FIG S6 Overexpression of SpoIID^Wch^ in C. trachomatis does not cause visible growth defects. C. trachomatis was transformed with a plasmid expressing SpoIID^Wch^ under the conrol of a tetracyclin-inducible promoter. (A) SpoIID^Wch^ is expressed only upon induction by tetracycline (aTC). SpoIID^Wch^ was detected by immunofluorescence using specific anti-SpoIID^Wch^ antibodies (green), concanavalin A (red), and DAPI (blue). Bar, 40 μm. (B) Morphology of RBs upon overexpression of SpoIID^Wch^ was observed using a C. trachomatis-specific anti-lipopolysaccharide (LPS) antibody (green). Bar, 1 μm. (C) Localization of SpoIID^Wch^ in C. trachomatis RBs. Cells were treated as described for panel A, and pictures were taken with same magnification as was used for panel B. (D) Quantification of sizes of the RBs shown in panel B was performed using the ImageJ software, and the results were analyzed by the use of GraphPad Prism software. Download FIG S6, PDF file, 0.2 MB. Copyright © 2019 Jacquier et al. 2019 Jacquier et al. This content is distributed under the terms of the Creative Commons Attribution 4.0 International license.

Since SpoIID^Wch^ localizes at the division septum, we can hypothesize that chlamydial SpoIIDs are involved in PG degradation at the division septum and that this degradation is linked to inner membrane invagination and thus could be involved in septation. Interestingly, SpoIID^Wch^ seems to degrade PG by two different mechanisms; we observed lytic transglycosylase activity on intact PG sacculi (resulting in anhydromuropeptides) and a muramidase activity on naked glycan chains (resulting mainly in nonanhydro glycan moieties). This potential bifunctional role of SpoIID^Wch^ as lytic transglycosylase and muramidase, which is not shared by SpoIID^Bsu^, might thus be involved in PG degradation with amidases, which are present in *Chlamydiales* (15, 16), and the two roles might thus together result in complete recycling of chlamydial PG, which is consistent with the detection of PG in dividing *Chlamydiae* only (10). However, SpoIID^Wch^ shows low activity on PG *in vitro*, indicating that it might require specific regulation in order to be fully active *in vivo*. Interestingly, we could show that SpoIID^Wch^ and AmiA^Wch^ can act together *in vitro* to digest PG. Further studies will now be required to better characterize the role of chlamydial SpoIID in division and to better understand how PG biosynthesis and PG-modifying enzymes such as NlpD or AmiA work together with RodZ and MreB during divisome formation and septation.

Nevertheless, proteins containing a SpoIID domain are not restricted to bacteria known to sporulate. Indeed, many different bacterial species, such as a large majority of cyanobacteria, possess SpoIID homologues (18). In contrast, many bacteria exhibiting a SpoIID homologue do not possess homologues of other important sporulation genes (Fig. S7). We thus assume, as was proposed previously by Morlot et al. (18), that SpoIID might degrade glycan chains in many different bacteria, including nonsporulating ones, and might do so even at the division septum. Data showing spread of the SpoIID domain in very diverse bacterial phyla support the idea of an ancestral role of the SpoIID domain in PG remodeling/degradation in cell division first and then (only later) a specialized role in spore formation in sporulating bacteria. Proteins containing this domain may thus constitute a component of a minimal bacterial division machine.

## MATERIALS AND METHODS

### Antibodies, probes, and reagents.

Polyclonal anti-Waddlia chondrophila rabbit antibodies were produced in-house as previously described (26). Unless otherwise indicated, reagents were purchased from Sigma-Aldrich (St. Louis, MO). Secondary Alexa Fluor 488 goat anti-mouse and 594 anti-rabbit antibodies originated from Molecular Probes (Grand Island, NY). SpoIID-coding genes containing point mutations as well as cyanobacterial SpoIID genes were synthetically produced by Integrated DNA Technologies (Coralville, IA). Strains and growth conditions are detailed in Text S1 in the supplemental material, and primers used in this study are listed in Table S2 in the supplemental material.

10.1128/mBio.01128-19.10 TABLE S2 Primers used in this study. Download Table S2, XLSX file, 0.01 MB. Copyright © 2019 Jacquier et al. 2019 Jacquier et al. This content is distributed under the terms of the Creative Commons Attribution 4.0 International license.

### Sequence alignments and signal sequence predictions.

Amino acid sequences of SpoIID homologues were downloaded from the NCBI website, aligned using MAFFT version v7.402 (27), and visualized using Jalview software v2 (28). Prediction of signal sequences and transmembrane domains was performed using SignalP v4.1 and the SignalP-noTM parameter (29) and TMHMM Server v. 2.0 (DTU Bioinformatics, Lyngby, Denmark).

### Cell culture and bacterial infection.

Vero cells (ATCC CCL-81) and McCoy (ATCC CRL-1696) cells were cultivated, and Vero cells were infected with W. chondrophila as described previously (14). Briefly, Vero cells or McCoy cells were grown in 75-cm^3^ flasks with 20 ml of Dulbecco’s modified Eagle’s medium (DMEM) supplemented with 10% fetal calf serum at 37°C and 5% CO_2_. Vero cells were harvested and counted. A cell suspension of 5 × 10^5^ cells/ml was allowed to adhere overnight. Vero cells were then infected with a 2,000× dilution of W. chondrophila in DMEM supplemented with fetal calf serum. Infection was synchronized by centrifugation at 1,790 × *g* for 15 min and incubation at 37°C for an additional 15 min. The medium was then removed, and the cell layer was washed once with phosphate-buffered saline (PBS) and further incubated in fresh medium at 37°C in a 5% CO_2_ atmosphere.

### UPLC and UPLC-QTOF peptidoglycan analysis.

PG was purified from the corresponding bacteria according to the protocol reported previously by de Jonge et al. (30) and as detailed in Text S1 in the supplemental material. The supernatants containing purified PG were adjusted to pH 9.0 with sodium borate and reduced with sodium borohydride for 20 min at room temperature. Finally, samples were adjusted to pH 3.5 with orthophosphoric acid and filtered with 0.45-μm-pore-size filters (Millipore, Billerica, MA) before injection into chromatographic system for analysis. Liquid chromatography was performed on a Waters Acquity UPLC H-class system (Waters, Milford, MA) equipped with a Waters Xevo G2-XS QTOF MS (Waters). Chromatographic separation was achieved on an Acquity UPLC BEH C_18_ column (Waters) (1.7 μm pore size, 150 mm by 2.1-mm inner diameter [id]). The mobile phase consisted of solvent A (0.1% formic acid–Milli-Q water) and solvent B (0.1% formic acid–acetonitrile). The gradient was set as follows: 0 to 3 min, 2% to 5% solvent B; 3 to 6 min, 5% to 6.8% solvent B; 6 to 7.5 min, 6.8% to 9% solvent B; 7.5 to 9 min, 9% to 14% solvent B; 9 to 11 min, 14% to 20% solvent B; 11 to 12 min, hold at 20% solvent B, keeping the constant flow of 0.250 μl/min; 12 to 12.1 min, 20% to 90% solvent B with a flow rate ramp of 0.250 μl/min to 0.300 μl/min, further holding the same flow rate and percentage of solvent B until 13.5 min; 13.5 to 13.6 min, 90% to 2% solvent B; 13.6 to 16 min, hold at 2% solvent B. The flow rate was shifted again to 0.250 μl/min at 16.1 min. Chromatographic column was equilibrated until 18 min for next analysis. Chromatographic data were recorded at 204 nm.

Mass spectrometry was performed on a Xevo G2-XS QTOF (Waters), which is a quadrupole time of flight mass spectrometer. The scan range ran from 100 to 2,000 *m/z*, with a scan time of 0.25 s. The instrument was operated in positive electrospray ionization mode. The capillary and sample cone voltages were 3 kV and 40 V, respectively. The gas flow rates were set at 100 and 500 liters/h for the cone gas and desolvation gas, respectively. The source temperature was 120°C, and the desolvation temperature was 350°C. For MS^E^, the low collision energy level was set at 6 eV, and the high collision energy level was ramped from 15 to 40 eV. A leucine-enkephalin reference was used at a concentration of 200 pg/ml as the lockmass, with a continuous flow rate of 5 μl/min and 0.25-s scan time, for an accurate analysis. All the processes involved in acquisition and analysis of data were controlled by Waters UNIFI software. Structural characterization of muropeptides was done on the basis of their MS and MS/MS fragmentation pattern data (31).

### Coimmunoprecipitation.

Vero cells infected or not infected with W. chondrophila were harvested 24 h p.i. and lysed by incubation in radioimmunoprecipitation assay (RIPA) buffer (Sigma) as described by the manufacturer. Lysates were clarified by centrifugation at 8,000 × *g* for 10 min at 4°C. Antibodies raised against RodZ of W. chondrophila (14) were bound to Dynabeads protein A beads as described by the manufacturer (Life Technologies). Cell lysate (1 ml) was incubated with the beads on a wheel for 30 min at room temperature, washed 3 times, and subjected to elution using Laemmli buffer (Alfa Aesar, Ward Hill, MA). The samples were then separated on SDS-PAGE, and bands were analyzed by mass spectrometry by the Protein Analysis Facility of the University of Lausanne.

### Immunofluorescence labeling and confocal microscopy.

Immunofluorescence labeling was performed as described previously (14). Vero cells were grown and infected on glass coverslips. They were then fixed by incubating 5 min in ice-cold methanol, washing 3 times with PBS, and incubating for at least 1 h in blocking buffer (PBS, 1% BSA, 0.1% saponin). Cells that adhered to the coverslip were then incubated with primary antibodies diluted in blocking buffer, washed three times with PBS, and incubated further with secondary antibodies and DAPI in blocking buffer. Coverslips were then washed 4 times with PBS and once with distilled water and mounted on glass slides with Mowiol (Sigma-Aldrich). Confocal microscopy images were obtained using a Zeiss LSM 510 Meta microscope (Zeiss, Oberkochen, Germany). Subsequent treatment and quantification of images were performed with ImageJ software.

### RNA extraction, cDNA synthesis, and qRT-PCR.

RNA quantification was performed as described earlier (14). Briefly, infected cells were harvested, and RNA was stabilized by addition of RNA Protect (Qiagen, Venlo, Netherlands) and incubation for 5 min at room temperature. The suspensions were then centrifuged at 5,000 × *g* for 10 min. The supernatant was removed, and pellet was kept at −80°C. RNA was extracted from the pellet using an RNeasy Plus kit (Qiagen). DNA was removed by selective digestion with DNase from an Ambion DNA-free kit (Thermo Fisher Scientific, Waltham, MA). cDNA was then synthesized by reverse transcription using a Goscript reverse transcription system (Promega), and *spoIID^Wch^* cDNA was quantified by qPCR performed on 4 μl of cDNA mixed with 10 μl of iTaq Universal SYBR green mix (Bio-Rad), 4.8 μl of water, and 0.6 μl each of primers SpoIID_RT_F and SpoIID_RT_R (Table S2) or 16S rRNA-specific primers WadF4 and WadR4 (32). qPCR was performed using a StepOne Plus real-time PCR system (Applied Biosystems, Waltham, MA) under the following conditions: 3 min of denaturation at 95°C followed by 45 cycles of 15 s of denaturation at 95°C and 1 min of annealing/elongation at 60°C.

### Peptidoglycan binding assay.

E. coli and B. subtilis PGs were extracted as described above. In the case of E. coli, samples were digested with pronase E (100 μg/ml)–10 mM Tris-HCl (pH 7.5) for 1 h at 60°C to remove Braun's lipoprotein. After addition of 1% (wt/vol) SDS, the reaction mixtures were heat inactivated and detergent was removed by washing in Milli-Q water. A 20-μl volume of purified PG was then incubated on ice with 2 μg of the indicated protein diluted in a total of 50 μl of PBS for 30 min. The pellet and supernatant were separated by ultracentrifugation. The pellet was washed once with PBS. Equivalent amounts of supernatant, wash, and pellet were separated by SDS-PAGE and detected by Western blotting (see Text S1).

### Peptidoglycan release assay.

The PG release assay was adapted from a method described previously by Morlot et al. (18). Briefly, E. coli PG was extracted as described above and digested overnight with α-amylase at 37°C. PG was then washed once with PBS, resuspended in 20 mM Remazol brilliant blue (RBB)–0.25 M NaOH, and incubated overnight at 37°C. The suspension was then neutralized with HCl, and excess dye was removed by repeated washes with PBS, to the point where the supernatant remained clear. A 40-μl volume of labeled PG was then incubated with 6 μg of the indicated proteins in a total of 100 μl PBS for 45 min at 37°C under conditions of agitation. PG was then sedimented by centrifugation at 16,000 × *g* for 10 min. The supernatant was collected, and its absorbance was measured at 595 nm using a Fluo Star Omega plate reader (BMG Labtech, Ortenberg, Germany).

### E. coli growth and morphology tests.

E. coli strains were grown overnight in LB medium at 37°C. Optical density at 600 nm (OD_600_) was measured by spectrophotometry. All the strains were diluted to an OD_600_ of 0.2 per ml and incubated 1 h at 37°C. IPTG was then added at 1 mM if indicated. Growth was measured by spectrophotometry 2, 4, and 6 h after the dilution. Microscopy was performed 6 h after dilution, using a 100× objective on an Axioplan 2 microscope (Zeiss). Lengths of bacteria were measured using ImageJ, and a minimum of 100 bacteria were classified as normal or elongated in duplicate in two independent experiments.
