# Supplementary material for: A SpoIID Homolog Cleaves Glycan Strands at the Chlamydial Division Septum
Source: mBio. 2019 Jul 16;10(4):e01128-19. doi: 10.1128/mBio.01128-19 (PMC6635528; doi:10.1128/mBio.01128-19)
Supplement: FIG S1 [file mBio.01128-19-sf001.pdf]

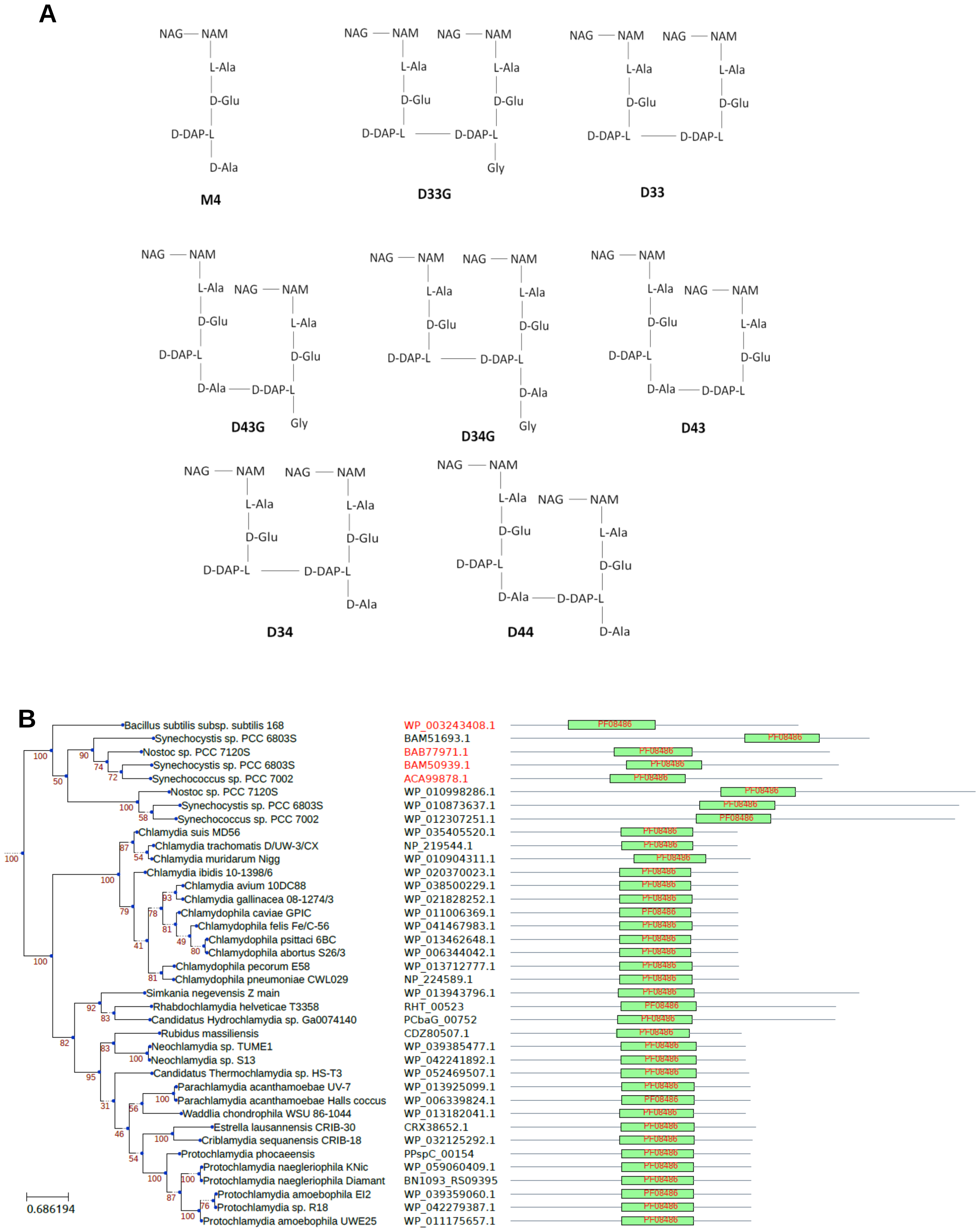
A
M4
D33G
D33
D43G
D34G
D43
D34
D44
NAG
NAM
L-Ala
D-Glu
D-DAP-L
D-Ala
Gly
B
Bacillus subtilis subsp. subtilis 168
Synechocystis sp. PCC 6803S
Nostoc sp. PCC 7120S
Synechocystis sp. PCC 6803S
Synechococcus sp. PCC 7002
Nostoc sp. PCC 7120S
Synechocystis sp. PCC 6803S
Synechococcus sp. PCC 7002
Chlamydia suis MD56
Chlamydia trachomatis D/UW-3/CX
Chlamydia muridarum Nigg
Chlamydia ibidis 10-1398/6
Chlamydia avium 10DC88
Chlamydia gallinacea 08-1274/3
Chlamydophila caviae GPIC
Chlamydophila felis Fe/C-56
Chlamydophila psittaci 6BC
Chlamydophila abortus S26/3
Chlamydophila pecorum E58
Chlamydophila pneumoniae CWL029
Simkania negevensis Z main
Rhabdochlamydia helveticae T3358
Candidatus Hydrochlamydia sp. Ga0074140
Rubidus massiliensis
Neochlamydia sp. TUME1
Neochlamydia sp. S13
Candidatus Thermochlamydia sp. HS-T3
Parachlamydia acanthamoebae UV-7
Parachlamydia acanthamoebae Halls coccus
Waddlia chondrophila WSU 86-1044
Estrella lausannensis CRIB-30
Criblamydia sequanensis CRIB-18
Protochlamydia phocaeensis
Protochlamydia naegleriophila KNic
Protochlamydia naegleriophila Diamant
Protochlamydia amoebophila EI2
Protochlamydia sp. R18
Protochlamydia amoebophila UWE25
0.686194
WP_003243408.1
BAM51693.1
BAB77971.1
BAM50939.1
ACA99878.1
WP_010998286.1
WP_010873637.1
WP_012307251.1
WP_035405520.1
NP_219544.1
WP_010904311.1
WP_020370023.1
WP_038500229.1
WP_021828252.1
WP_011006369.1
WP_041467983.1
WP_013462648.1
WP_006344042.1
WP_013712777.1
NP_224589.1
WP_013943796.1
RHT_00523
PCbaG_00752
CDZ80507.1
WP_039385477.1
WP_042241892.1
WP_052469507.1
WP_013925099.1
WP_006339824.1
WP_013182041.1
CRX38652.1
WP_032125292.1
PPspC_00154
WP_059060409.1
BN1093_RS09395
WP_039359060.1
WP_042279387.1
WP_011175657.1
PF08486

Figure S1
