# Supplementary material for: A SpoIID Homolog Cleaves Glycan Strands at the Chlamydial Division Septum
Source: mBio. 2019 Jul 16;10(4):e01128-19. doi: 10.1128/mBio.01128-19 (PMC6635528; doi:10.1128/mBio.01128-19)
Supplement: TEXT S1 [file mBio.01128-19-s0001.docx]

**Supplementary data:**

**Supplementary methods:**

**Strains and growth conditions**

*Escherichia coli* EC251, BL21 and *Bacillus subtilis* were grown in Luria-Bertani broth (LB). SPOR mutants were kindly provided by Prof. David Weiss (University of Iowa, Iowa City, IA).

*Waddlia chondrophila* (ATCC VR-1470T) was grown in co-culture with *Acanthamoeba castellanii* (ATCC 30010) in Peptone-Yeast extract-Glucose medium incubated at 28°C for 6 days. Bacterial suspension was then filtered using a 5 μm syringe filter to remove intact amoebae and amoebal debris. The flow-through was used to infect mammalian cells.

DNA of *W. chondrophila* was extracted from purified EBs as described. DNA from *C. trachomatis* LGV strain (ATCC VR-902B) was kindly provided by Prof. Ken Fields (Lexington, KY).

**DNA extraction and quantitative PCR**

50 μl of a suspension of infected cells were taken at the indicated time points. DNA was extracted as previously described {Jacquier, 2014 #65}, using the DNA Wizard SV Genomic DNA purification system (Promega) and eluted with 200 μl of water. qPCR was performed as described {Goy, 2009 #41}, using 5 μl of extracted DNA, 200 nM of primers WadF4 and WadR4 and 100 nM of the WadS2 probe in iTaq Supermix with ROX (Bio-Rad). qPCR was performed using a stepOne Plus Real-time PCR system (Applied Biosystems) as follows: 3 min at 95°C followed by 40 cycles of 15 sec at 95°C and 1 min at 60°C.

***C. trachomatis* transformation**

*C. trachomatis* were transformed as described by Mueller et al. (1). Briefly, *C. trachomatis* EBs were incubated with 50 mM CaCl_2_ and 12 μg of the plasmid to transform for 30 min at room temperature. McCoy cells were then infected with this suspension by centrifugation for 1 hour at 900 x *g*. Cells were then incubated for 7 hours at 37°C, 5% CO_2_ before addition of the antibiotic (0.6 μg/ml penicillin). Infected cells were harvested by scraping every two days and passed on fresh McCoy cells until appearance of inclusions.

**Peptidoglycan isolation**

Gram-negative bacteria

Vero cells infected with *W. chondrophila* were harvested 24 h p.i., incubated with lysis solution (1% Basic phenol, 19% ethanol, 0.4% SDS, 10 µg/ml DNase) for 30 min at 4°C. The plates were then scraped, the cell suspension was transferred to centrifuge tubes and centrifuged at 25’000 x *g* for 60 min at 4ºC. The obtained pellet was re-suspended in a minimal volume of PBS.

*E. coli* was grown overnight in LB medium and harvested by centrifugation. Bacteria were lysed as described for *W. chondrophila*.

The samples were then slowly dropped into an equal volume of boiling 10% (wt/vol) SDS and vigorously stirred for at least 2 h and left stirring overnight. The insoluble fractions were recovered by high speed centrifugation (150’000 x *g*, 15 min, 25°C) and washed until the fractions were free from SDS. Samples were digested with pronase E (100 μg/ml) in 10 mM Tris-HCl, pH 7.5, for 1 h at 60°C to remove Braun's lipoprotein. After addition of 1% SDS (w/v), reaction mixtures were heat inactivated and detergent was removed by washing in Milli-Q water.

Gram-positive bacteria:

Overnight culture of *B. subtilis* grown in LB media was harvested by centrifugation and re-suspended in PBS. The samples were then slowly dropped into an equal volume of boiling 10% SDS (w/v) and vigorously stirred for at least 2 h and left stirring overnight. The insoluble fractions were recovered by high speed centrifugation (150’000 x *g*, 15 min, 25°C) and washed until the fractions were free from SDS.

**Protein extraction, SDS-PAGE and Western blotting**

Cell lysate or purified proteins were resuspended in Laemmli sample buffer and were separated by SDS-PAGE at 30 mA per gel using 12% polyacrylamide precast gels (BioRad). Gel was then either stained with Coomassie or further processed by electroblotting. In that case, proteins were transferred on nitrocellulose membrane (GE Healthcare) by electroblotting for 1 h at 75 mV. The membrane was then blocked with 5% defatted milk (Applichem) and incubated for 2 h with a primary antibody dilution. The membrane was then washed three times and incubated with a secondary antibody linked to horseradish peroxydase (BioRad) for 1 h. The membrane was finally washed three times and luminescence was detected with the help of an Amersham ECL Western Blot Detection Reagent kit using an ImageQuant LAS4000 (GE healthcare).

**SpoIID^Wch^ expression, purification and antibodies production**

The sequence coding for SpoIID^Wch^ lacking its N-terminal transmembrane domain (SpoIID^Wch^-TMD) was amplified by PCR from *W. chondrophila* genomic DNA using prolonged primers wchSpoIID_attB1 and wchSpoIID_attB2 (Table S2) for cloning using the Gateway system (Thermo Fisher Scientific). A similar approach was performed to amplify the gene coding for SpoIID^Bsu^ lacking its N-terminal transmembrane domain, using the primers bsuSpoIID_attB1 and bsuSpoIID_attB2 (Table S2). Following the manufacturer’s instructions, the PCR products were then cloned in a pDONR201 plasmid with the help of a LR clonase. Sequence integrity of the insert was controlled by sequencing. The inserts were then recombined with a pET15bGw plasmid (allowing their expression in fusion with an N-terminal 6xHis tag) using a BP clonase. Correct insertion was controlled by restriction and the resulting plasmids were transformed into BL21 strain for protein expression. The BL21 strain containing the pET15bGw-SpoIID^Wch^-TMD was grown overnight, diluted and grown to an OD_600_ of 0.5/ml. IPTG (Applichem, Darmstadt, Germany) was then added to induce overexpression of a 6xHis-tagged version of the proteins. 6 hours later, bacteria were harvested by centrifugation at 4’000 x *g* at 4°C for 10 minutes. The pellet was lysed by resuspension and shaking for 30 min in lysis buffer containing 1x Fastbreak Reagent (Promega), 20 U/ml DNAse I (Biolabs), 0.2 mg/ml lysosyme and 1x Protease inhibitor cocktail (Promega). The lysate was then centrifuged at 20’000 x *g* for 5 minutes to remove cellular debris and insoluble proteins. SpoIID^Wch^ was purified from supernatant by incubation with Magnetic beads (Promega). Beads were then washed 3 times with wash buffer and protein was eluted from beads by incubation with elution buffer (100 mM HEPES, 500 mM imidazole). Proteins were concentrated and imidazole was removed using Amicon columns (Millipore). The purified proteins were quantified by Bradford (Bio-Rad) and kept at 4°C less than a week before their use in in vitro studies. SpoIID^Wch^ was also used for mice immunization (Eurogentec).

**Phylogeny of SpoIID homologs**

SpoIID homologs encoded in the genome of *Chlamydiae* spp., *B. subtilis*, *Nostoc* sp. PCC 7120, *Synechocystis* sp. PCC 6803 and *Synechococcus* sp. PCC 7002 were identified based on the identification of the Pfam domain PF08486 with interproscan version 5.30-69.0 (2). Amino acid sequences were aligned with MAFFT v7.271 (3). The phylogeny was reconstructed with RAxML (4) with the PROTGAMMALG model and 100 boostrap replicates. The figure was drawn with the ete3 toolkit (5).

**Taxonomic range of the SpoIID domain**

Coding sequences of the 6661 reference and representative genomes available from RefSeq (September 2017) were downloaded from RefSeq ftp (ftp://ftp.ncbi.nlm.nih.gov/refseq/). Hidden Markov models of the PFAM database (6) were used to search for sporulation-related domains using hmmsearch (HMMER version 3.1b2, (7)). Hits were filtered based on PFAM trusted cutoffs. Genomes were then grouped by order based on the NCBI Taxonomy database (8) with the python library ete2. Only orders exhibiting a minimum of 5 genomes are reported on Fig. S6.

**Supplementary references:**

1. Mueller KE, Wolf K, & Fields KA (2017) Chlamydia trachomatis Transformation and Allelic Exchange Mutagenesis. *Curr Protoc Microbiol* 45:11A 13 11-11A 13 15.

2. Jones P*, et al.* (2014) InterProScan 5: genome-scale protein function classification. *Bioinformatics* 30(9):1236-1240.

3. Katoh K & Standley DM (2013) MAFFT multiple sequence alignment software version 7: improvements in performance and usability. *Mol Biol Evol* 30(4):772-780.

4. Stamatakis A (2014) RAxML version 8: a tool for phylogenetic analysis and post-analysis of large phylogenies. *Bioinformatics* 30(9):1312-1313.

5. Huerta-Cepas J, Serra F, & Bork P (2016) ETE 3: Reconstruction, Analysis, and Visualization of Phylogenomic Data. *Mol Biol Evol* 33(6):1635-1638.

6. Finn RD*, et al.* (2016) The Pfam protein families database: towards a more sustainable future. *Nucleic Acids Res* 44(D1):D279-285.

7. Eddy SR (2011) Accelerated Profile HMM Searches. *PLoS Comput Biol* 7(10):e1002195.

8. Federhen S (2012) The NCBI Taxonomy database. *Nucleic Acids Res* 40(Database issue):D136-143.
