# Supplementary figures and images for: A SpoIID Homolog Cleaves Glycan Strands at the Chlamydial Division Septum
Source: mBio. 2019 Jul 16;10(4):e01128-19. doi: 10.1128/mBio.01128-19 (PMC6635528; doi:10.1128/mBio.01128-19)

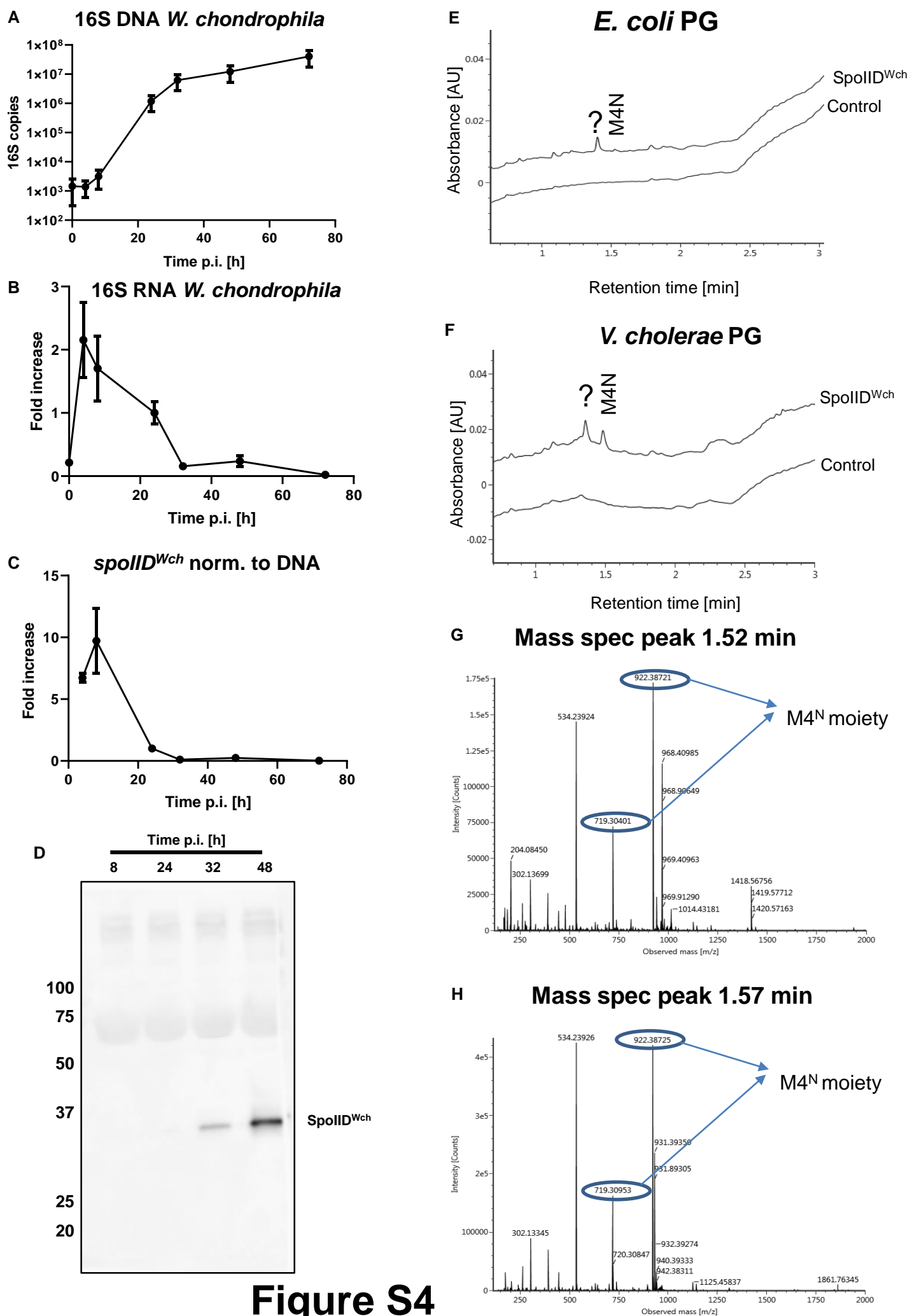

Supplement: FIG S4 [file mBio.01128-19-sf004.pdf]

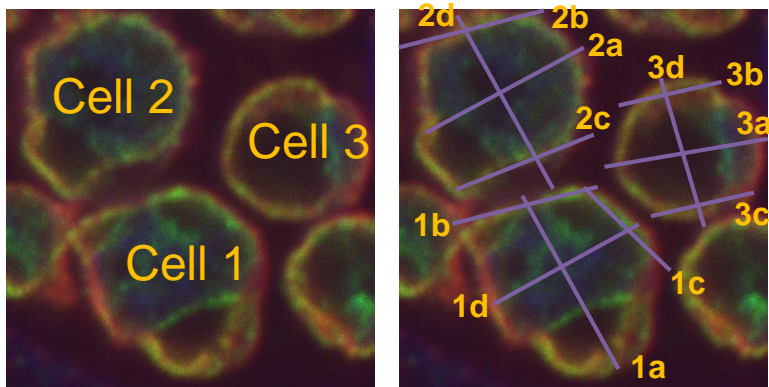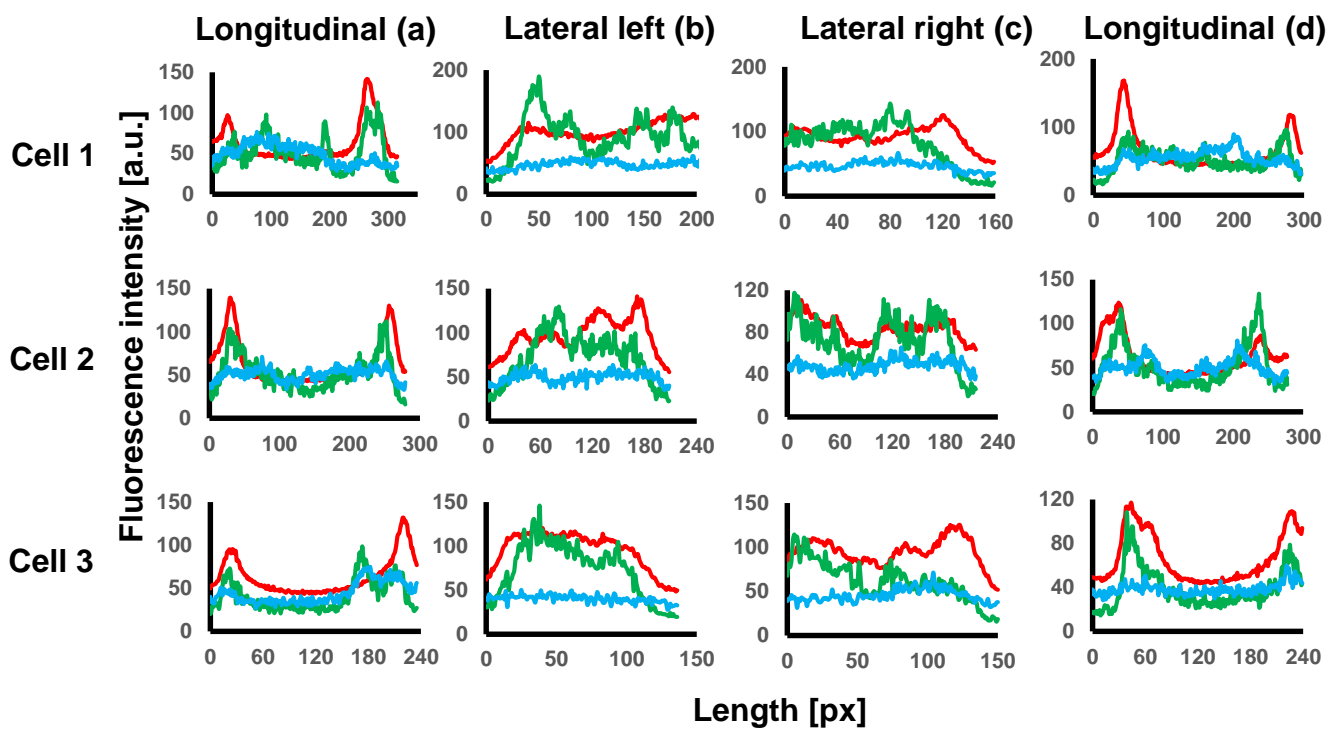

**Figure S3**

Supplement: FIG S3 [file mBio.01128-19-sf003.pdf]

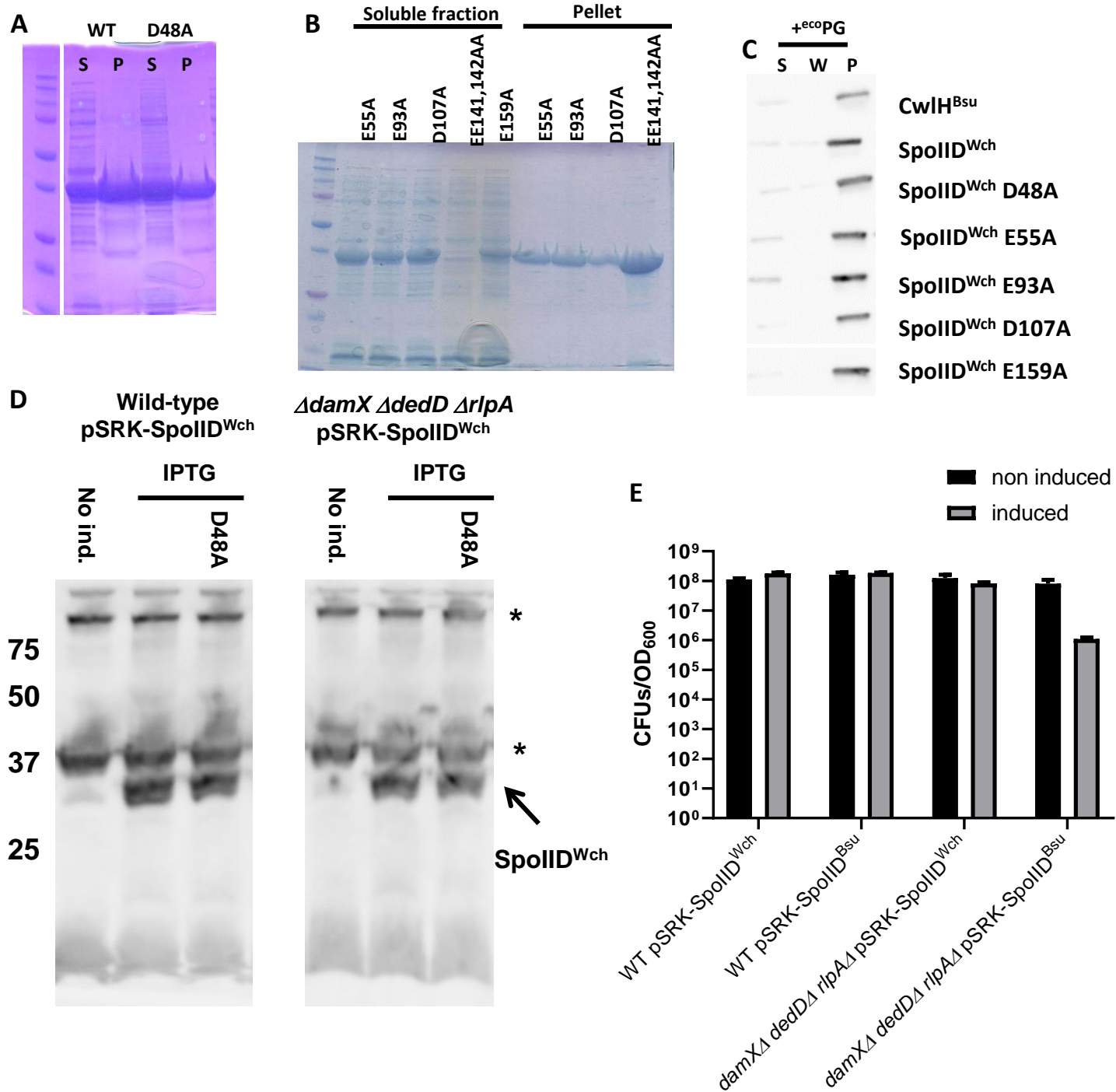

**Figure S5**

Supplement: FIG S5 [file mBio.01128-19-sf005.pdf]

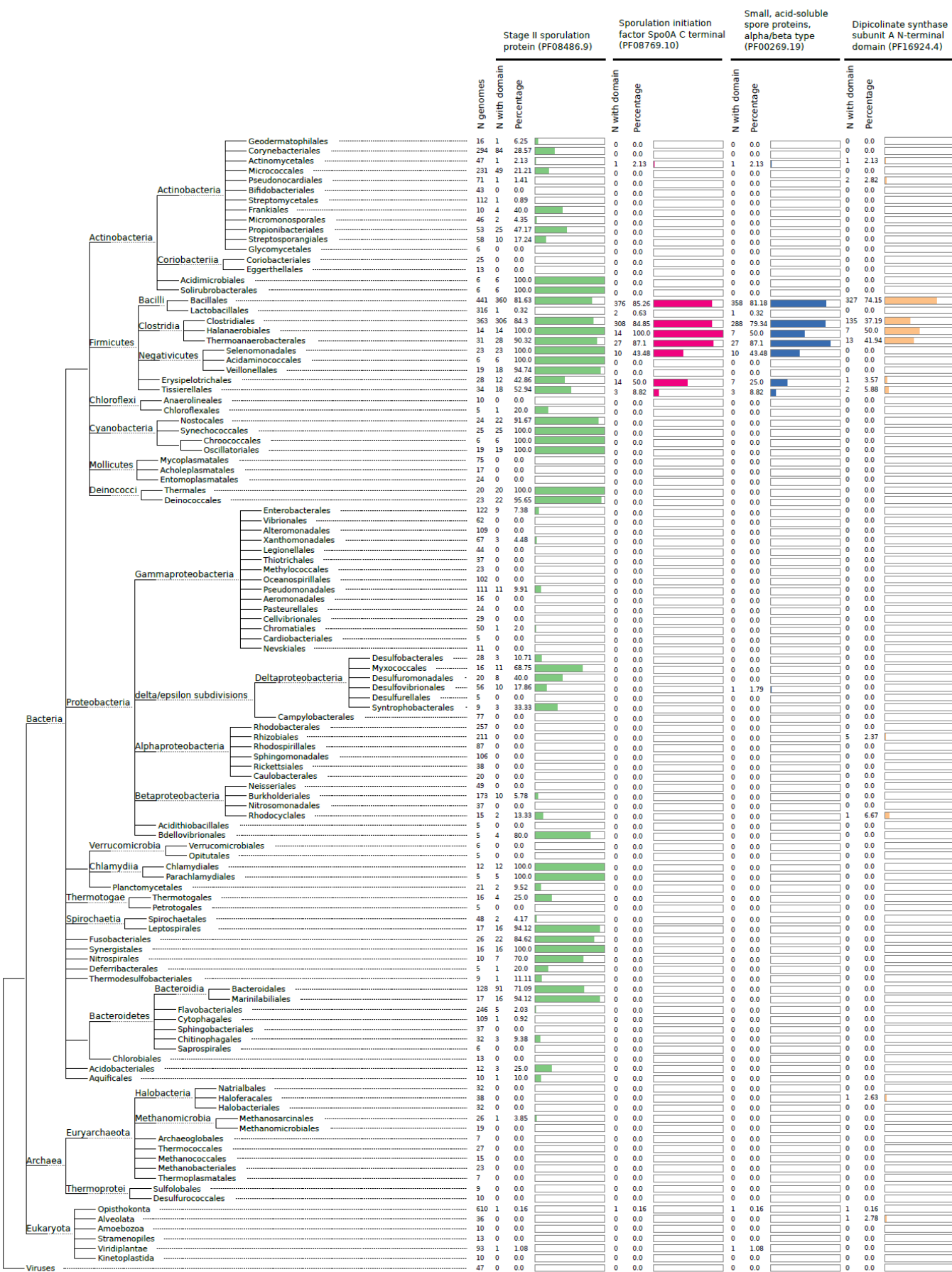

Figure S7

Supplement: FIG S7 [file mBio.01128-19-sf007.pdf]
