# Supplementary material for: A SpoIID Homolog Cleaves Glycan Strands at the Chlamydial Division Septum
Source: mBio. 2019 Jul 16;10(4):e01128-19. doi: 10.1128/mBio.01128-19 (PMC6635528; doi:10.1128/mBio.01128-19)
Supplement: FIG S2 [file mBio.01128-19-sf002.pdf]

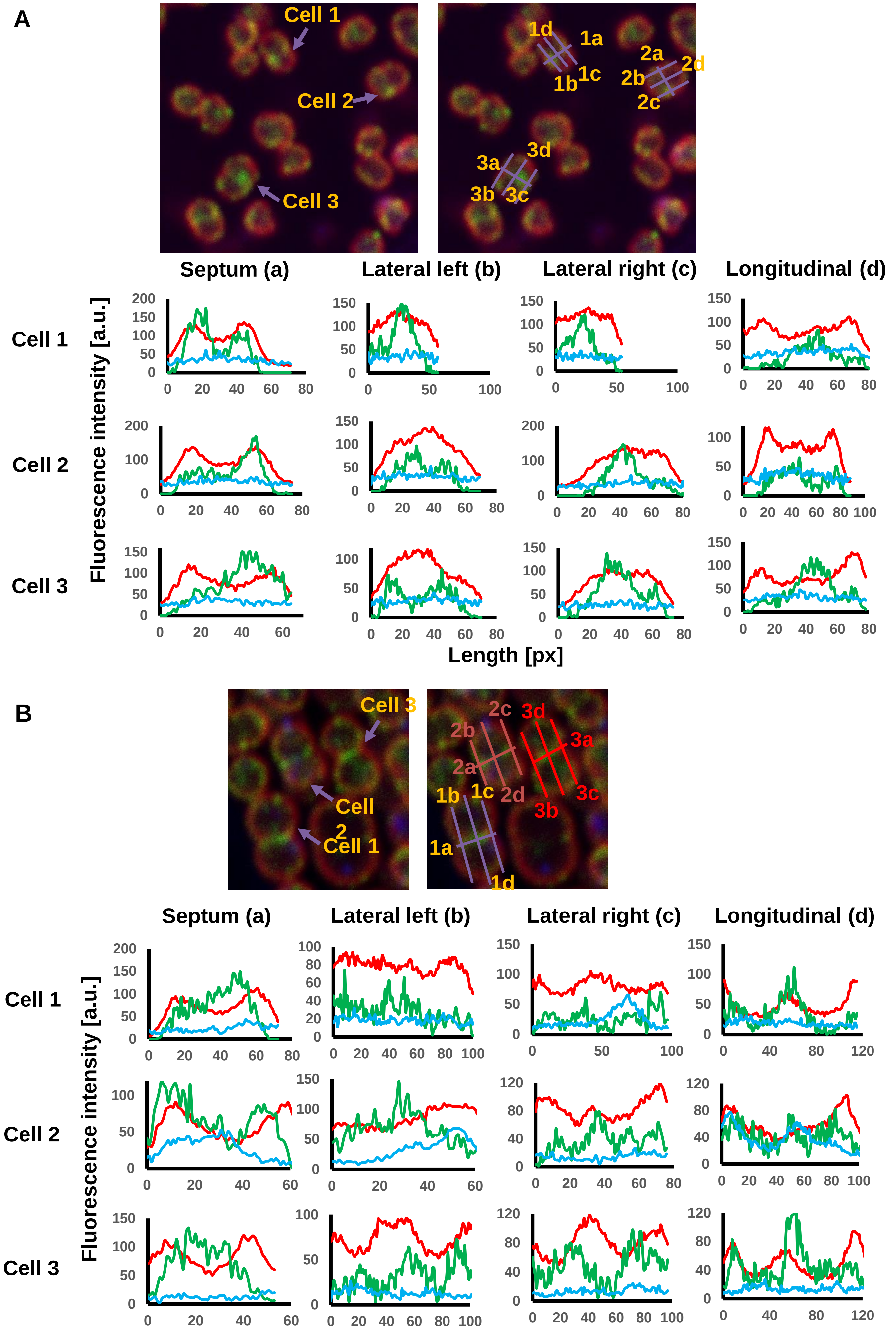
A
Cell 1
Cell 2
Cell 3
1a
1b
1c
1d
2a
2b
2c
2d
3a
3b
3c
3d
Septum (a)
Lateral left (b)
Lateral right (c)
Longitudinal (d)
Fluorescence intensity [a.u.]
Length [px]
B
Cell 3
Cell 2
Cell 1
Septum (a)
Lateral left (b)
Lateral right (c)
Longitudinal (d)
Fluorescence intensity [a.u.]
Length [px]

Figure S2
