# Supplementary material for: A SpoIID Homolog Cleaves Glycan Strands at the Chlamydial Division Septum
Source: mBio. 2019 Jul 16;10(4):e01128-19. doi: 10.1128/mBio.01128-19 (PMC6635528; doi:10.1128/mBio.01128-19)
Supplement: FIG S6 [file mBio.01128-19-sf006.pdf]

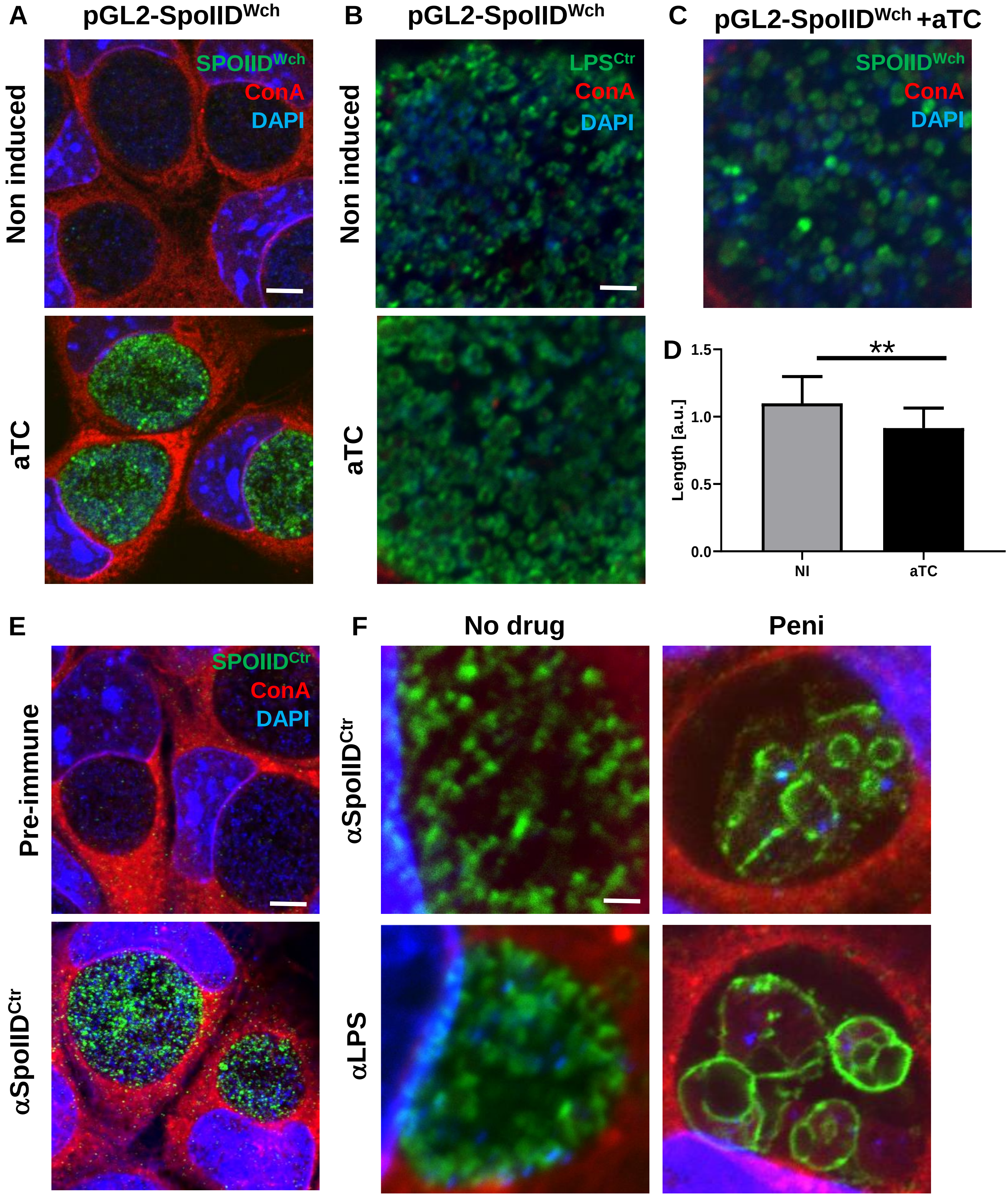
A
pGL2-SpoIIDWch
Non induced
SPOIIDWch
ConA
DAPI
aTC
B
pGL2-SpoIIDWch
LPSCtr
ConA
DAPI
Non induced
aTC
C
pGL2-SpoIIDWch +aTC
SPOIIDWch
ConA
DAPI
D
Length [a.u.]
1.5
1.0
0.5
0.0
**
NI
aTC
E
SPOIIDCtr
ConA
DAPI
Pre-immune
αSpoIIDCtr
F
No drug
Peni
αSpoIIDCtr
αLPS

Figure S6
